# Decadal East Asian monsoon anomalies and implications for societal conflicts

**DOI:** 10.1126/sciadv.aee1648

**Published:** 2026-05-06

**Authors:** Kan Zhao, Yijia Liang, Changchun Huang, Yongjin Wang, Weiyi Sun, Hai Cheng, Jianjun Yin, Di Hu, Mi Yan, Liang Ning, Zhaoyuan Yu, R. Lawrence Edwards

**Affiliations:** ^1^ State Key Laboratory of Climate System Prediction and Risk Management; and Key Laboratory for Virtual Geographic Environment of Ministry of Education, Jiangsu Center for Collaborative Innovation in Geographical Information Resource Development and Application, Nanjing 210023, China.; ^2^ School of Geography, Nanjing Normal University, Nanjing 210023, China.; ^3^ Institute of Global Environmental Change, Xi’an Jiaotong University, Xi’an 710049, China.; ^4^ Key Laboratory of Karst Dynamics, Ministry of Natural Resources and Guangxi; and Institute of Karst Geology, Chinese Academy of Geological Sciences, Guilin 541004, China.; ^5^ Department of Geology and Geophysics, University of Minnesota, Minneapolis, MN 55455, USA.

## Abstract

The East Asian summer monsoon anomalies, responsible for floods and droughts across China, pose great challenges to the climate resilience of human society. However, mechanisms and impacts of decadal-scale monsoon variability remain poorly understood due to limited instrumental data. Our annual-resolution speleothem record (1787–2007 CE) from China, along with simulation results, reveals a persistent monsoon weakening since the end of the Little Ice Age, superimposed by decadal oscillations. Five monsoon extremes, triggered by diminished solar output and further amplified by ocean-atmosphere processes in the Atlantic and Pacific oceans, caused widespread megadroughts in China. One marked flood-drought abrupt alternation in the 1850s substantially contributed to the Taiping Rebellion (1851–1864 CE). Our findings demonstrate that monsoon anomalies can cause crop failure and exacerbate overpopulation, ultimately leading to social unrest, particularly in regions experiencing rapid demographic expansion.

## Teaser

Extreme flood-drought swings triggered crop shortage and overpopulation, fueling the rise of the Taiping Rebellion.

## INTRODUCTION

The East Asian summer monsoon (EASM) plays a critical role in transporting moisture to the most densely populated regions of the Asian continent and deeply affects agriculture, water resources, and socioeconomic activities in China. Instrumental data indicate that the EASM has undergone a persistent weakening since the mid-20th century, resulting in deficient summer rainfall in northern China and excessive rainfall in southern China (*1*–*3*). Possible drivers of the EASM weakening include the increasing anthropogenic aerosol emissions (*4*, *5*), cooling over the Tibetan Plateau (*1*, *2*), warming of tropical oceans (*3*, *6*), and the decline in the Atlantic meridional overturning circulation (AMOC) (*7*). Superimposed on the long-term trend are decadal EASM variabilities, lending to two meridional modes of summer precipitation in China: the dipole pattern (e.g., southern flooding and northern drought) and the tripolar pattern (e.g., drying in the south and north but flooding in the Yangtze River Basin). The decadal variations are likely associated with solar activity (*8*, *9*), the Pacific decadal oscillation (PDO) (*4*, *10*), the Atlantic multidecadal oscillation (AMO) (*3*, *4*), and/or tropical volcanic eruptions (*11*, *12*). However, the underlying mechanism of the observed EASM changes, particularly on decadal timescales, remains ambiguous, partly due to uncertainties in the magnitude of the anthropogenic influence, as well as due to the interplay between internal variability and external forcing (*4*).

Proxy evidence and model simulations indicate that EASM anomalies and protracted floods/droughts recurred in the historical period and caused profound impacts on ancient Chinese civilizations (*5*, *13*–*16*). For example, megadroughts during the “Late-Ming Weak Monsoon Periods” (1580–1660 CE) (*5*) resulted in declined grain yields and widespread famine, ultimately contributing to peasant uprisings and the collapse of the Ming Dynasty (*5*, *15*). The Taiping Rebellion (1851–1864 CE), regarded as the deadliest civil war in Chinese history, has been associated with a cooling trend in 19th-century China, accompanied by increased flooding and other extreme climate events (*14*, *16*). Cooling and flooding adversely affected grain yields, exacerbating the human-land conflicts during the Qing Dynasty. A recent study indicates that floods influence local armed conflicts, often leading to rebellions (*17*). However, the detailed processes of how extreme climate affects human society are not yet fully understood, necessitating continuous high-resolution climate reconstructions to elucidate them.

Recent studies indicate that precipitation variability on daily-to-multiyear timescales increases under the scenario of global warming, thereby inducing large-amplitude wet-dry swings (*18*, *19*). The frequency of flood-drought abrupt alteration has increased more than threefold during 1979–2022, causing substantial damage to society and the ecosystem (*20*). However, the dynamics and impacts of historical drought-flood abrupt alternation remain poorly understood due to the limited temporal resolution and chronological uncertainties in proxy records. In this study, we present an annual resolution δ^18^O record from an annually laminated speleothem (DG7) spanning the past two centuries from Daoguan Cave (26°30′N, 105°50′E, 1420 m above sea level; fig. S1), southwestern China, to explore the dynamics and effects of the decadal EASM variability. We first present robust evidence indicating that Chinese cave δ^18^O records along the moisture transport pathway from the Indian Ocean (fig. S1) demonstrate a spatially coherent EASM variability, strongly correlated with precipitation changes in northern China. The decadal EASM variability is related to the 11-year solar cyclicity, modulated by the AMOC, PDO, and AMO. We then investigate the connection between the decadal EASM anomalies and the Taiping Rebellion, suggesting that flood-drought abrupt alteration causes agricultural collapse and extreme overpopulation, ultimately contributing to social instability.

## RESULTS

### Chronology

The DG7 sample was actively growing when collected in 2007 CE (hereafter, CE is omitted to facilitate the flow of the text). The results of x-ray diffraction (XRD) analyses suggest that this stalagmite is composed of calcite minerals (Materials and Methods) (fig. S2). Laminations above the depth of 130.5 mm consist of couplets of white-porous calcites and dark-compact calcites (figs. S2 and S3). These paired white and dark laminae resemble the annual laminations described previously (*21*). Further evidence of annual layer determination is the correspondence between annual cycles in the DG7 δ^13^C record and the paired white and dark laminae; that is, relatively positive δ^13^C values are associated with the dark laminae and more negative values with the white laminae (fig. S3). A total of 220 annual layers were counted to a depth of ~130.5 mm, with a cumulative error of 4 years.

**Fig. 1.**
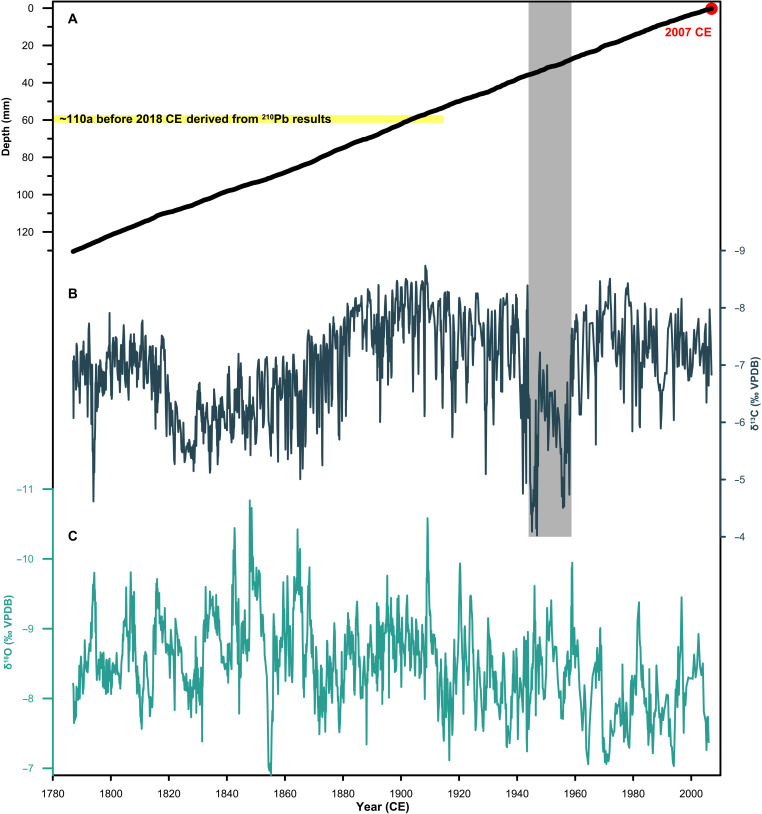
Chronology of DG7 stalagmite. (**A**) Age model based on annual layer counting, referring to the top as 2007 CE, when the stalagmite was actively growing at the time of collection (red dot). The shaded yellow rectangle indicates the age constraint from ^210^Pb dating results. (**B**) δ^13^C time series with distinct annual cycles. The shaded gray rectangle indicates two anomalies in δ^13^C values centered in 1945 and 1958 CE (**C**) δ^18^O time series. 110a, 110 years; VPDB, Vienna Pee Dee Belemnite.

The age model of DG7 is established on the basis of annual layer counting, with the top layer assigned to 2007 (Fig. 1A). Despite large age uncertainties for ^230^Th dating (Materials and Methods) (table S1), our layer-counting age model is supported by two anchors. First, the ^210^Pb dating results (Materials and Methods) (table S2) suggest that the carbonate deposits located above a depth of ~60 mm are younger than 110 years before present (Fig. 1A and fig. S4), which is consistent with our age model. Second, two anomalies in δ^13^C values centered on 1945 and 1958, with amplitudes of ~4.4 per mil (‰) (Fig. 1B), are related to the well-documented large-scale deforestation (supplementary text 1 and fig. S5). Sedimentary records from nearby plateau lakes demonstrate two notable peaks in biomass burning during 1940–1960 (*22*, *23*) and align closely with the DG7 δ^13^C record (fig. S6). Therefore, we conclude that the layer-counting timescale from 1787 to 2007 is the most accurate estimate of the DG7 chronology.

### DG7 δ^18^O time series and iCESM-LME

The δ^18^O record covers the past two centuries from 1787 to 2007, with a sampling resolution of ~0.15 years on average (Fig. 1C) (Materials and Methods). The δ^18^O values vary from −10.84 to −6.90‰, with an average value of −8.51‰. The δ^18^O record exhibits a decreasing trend from 1787 until ~1850, followed by a long-term increasing trend that persists to the present. A series of decadal- to interannual-scale fluctuations, with amplitudes of 1 to 2‰, are superimposed on the long-term trend (Fig. 1C). Spectral analysis reveals notable periodicities at ~14 and 1 to 3 years (fig. S7). The Hendy test (fig. S8) (*24*) and good replication of well-dated stalagmite δ^18^O records (fig. S9) indicate that calcite δ^18^O variations are little affected by kinetic fractionation, thus primarily reflecting climate changes (*25*).

The simulation results were derived from the Community Earth System Model Last Millennium Ensemble (CESM-LME) (*26*) and isotope-enabled Last Millennium simulations (iCESM-LME) (*27*) (Materials and Methods). The simulated annual precipitation δ^18^O, annual precipitation, and summer precipitation in northern China (105°E to 120°E, 35°N to 42°N) over the past two centuries are compared with the DG7 δ^18^O record to better interpret the proxy record.

## DISCUSSION

### A robust EASM record derived from stalagmite δ^18^O records

The speleothem δ^18^O has been interpreted as a proxy of the EASM intensity (*28*, *29*) based on two mechanisms, i.e., changes in the fraction of monsoon rainfall in annual totals (*30*) and changes in the amount of rainout between tropical sources and cave sites (*31*). Here, we use the DG7 δ^18^O record in conjunction with published data in the monsoon region of China to further investigate this issue. Cave δ^18^O records from southwestern [Daoguan and Dongge Cave (*32*)], central [Heshang Cave (*33*)], and northern China [Shihua Cave (*6*) and Qujia Cave (*7*)] exhibit a coherent variability, especially on the long-term trend (fig. S9, A to D). Correlation analyses also support a broad similarity among these δ^18^O records (table S3). Besides, the decadal oscillations observed in these δ^18^O records are largely comparable. The discrepancies between them are attributable to two main factors: spatial heterogeneities in hydroclimate (e.g., local precipitation and evapotranspiration) and differential isotopic signal smoothing due to site-specific karst processes. It is noteworthy that these caves are situated along the southwestern pathway of moisture transport in the monsoon region of China, with a dominant moisture source from the Indian Ocean (fig. S1). These observations support the remote-depletion effect originating from the Indian Ocean (*31*, *34*, *35*), which is consistent with previous modeling results (*36*, *37*). Therefore, the DG7 δ^18^O primarily reflects the EASM intensity, with lower δ^18^O values corresponding to stronger EASM intensity and more moisture transport from the Indian Ocean.

**Fig. 2.**
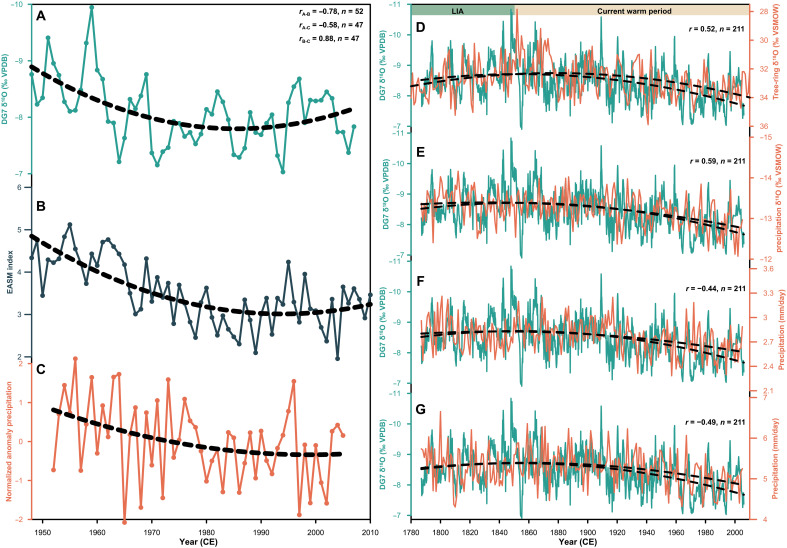
Climatic interpretation of stalagmite δ^18^O. Left: Comparison of the annually resolved DG7 δ^18^O record (**A**), observed EASM intensity index from 1948 to 2017 (*3*) (**B**), and normalized anomaly series of summer precipitation across North China (*38*) (**C**). Right: Comparison of the DG7 δ^18^O record, tree-ring δ^18^O record (*39*) (**D**), simulated annual precipitation δ^18^O (**E**), annual precipitation (**F**), and summer precipitation (**G**) in northern China (35°N to 42°N, 105°E to 120°E). DG7 record is the aquamarine line. Modeling results are from iCESM-LME (*27*). The black dashed lines indicate a second-order polynomial fitting for all records. The correlation coefficient is calculated with a 1-year equal interval interpolation and a 9-year moving average (significant at the *P* < 0.01 level). VSMOW, Vienna Standard Mean Ocean Water.

Multiple lines of evidence support our interpretation of stalagmite δ^18^O. First, the high-resolution DG7 δ^18^O record demonstrates a strong correlation with the observed EASM index (*3*) and summer precipitation in northern China (*38*) (Fig. 2, A to C), suggesting that enhanced (weakened) EASM intensity leads to increased (decreased) rainfall in northern China (*1*–*3*). Second, the DG7 δ^18^O record displays a high degree of similarity with a tree-ring δ^18^O record from the northern fringe of the EASM region (Fig. 2D) (*39*) and the simulated precipitation δ^18^O in northern China (Fig. 2E) (*27*), confirming the spatial coherence of the EASM variability (*36*, *37*). Third, the DG7 δ^18^O record is strongly correlated with the simulated annual (Fig. 2F) and summer (Fig. 2G) precipitation in northern China (*27*), consistent with the meteorological observations (Fig. 2, A to C). Fourth, Chinese cave δ^18^O records along the moisture transport pathway from the Indian Ocean vary broadly similar to summer rainfall of the Indian subcontinent during 1901–2007 (fig. S9) (*40*), supporting the remote-depletion effect (*34*–*37*). Last, the DG7 δ^18^O-based EASM reconstruction exhibits a negative correlation with the regional hydrological record in southwestern China (supplementary text 2 and fig. S10), consistent with the modern precipitation dipole pattern. In conclusion, aligning with previous meteorological (*1*–*3*, *38*) and modeling (*36*, *37*) evidence, we confirm that the DG7 δ^18^O record is a reliable proxy of EASM intensity, with an intensified EASM (lighter δ^18^O) resulting in increased monsoon rainfall over northern China.

Notably, we identify a prominent weakening trend in EASM since the end of the Little Ice Age (LIA; ~1850 CE; Fig. 2 and fig. S11A). Although the EASM variations are typically positively correlated with changes in Northern Hemisphere temperatures on various timescales (*5*, *13*, *28*), this relationship has reversed since 1850 (fig. S11, A and B), suggesting a substantial anthropogenic influence on monsoon dynamics. Previous Coupled Model Intercomparison Project Phase 5 (CMIP5) models indicate that the observed decreasing trend in global land monsoon precipitation (1948–2005) is attributed to anthropogenic forcing, notably to anthropogenic aerosol forcing (*41*). Here, attribution analysis using the CESM-LME (*26*) reveals a precipitation decline in northern China since 1850 in both the all-forcing and aerosol-only simulations, in contrast to an increase in the greenhouse-gas-only simulation (fig. S11, D to F). This finding aligns with the Sixth Assessment Report of the Intergovernmental Panel on Climate Change (*4*), which attributes the 20th-century EASM decline and precipitation reduction to anthropogenic aerosol-induced cooling. The cooling effects of aerosol forcing are twofold: a thermodynamic effect due to the reduction in atmospheric humidity, and a dynamic effect due to the weakening of the land-sea thermal contrast and thus monsoon circulation. Furthermore, increasing aerosol emissions in the Northern Hemisphere have likely driven a southward movement of the Intertropical Convergence Zone (ITCZ) since 1850 (*42*), thereby weakening EASM circulation. These findings underscore that controlling aerosol emissions is critical to mitigating future monsoon rainfall declines.

### Decadal EASM variabilities and their dynamics

**Fig. 3.**
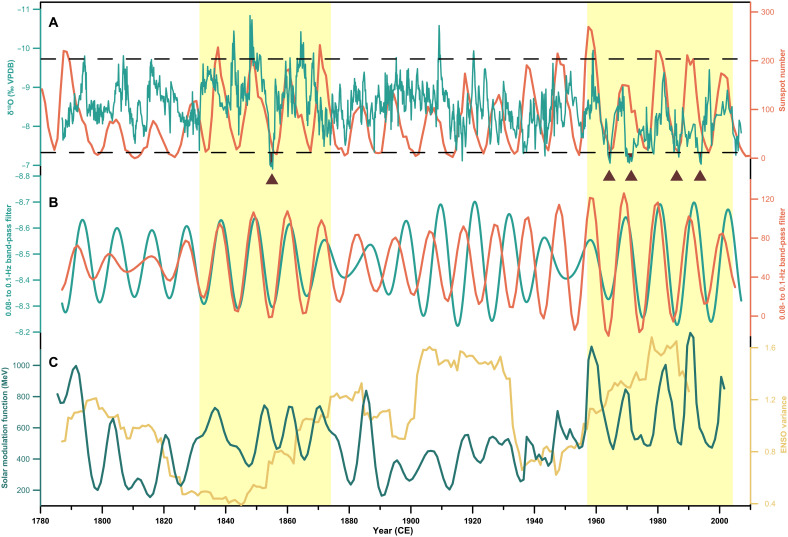
Solar forcing on decadal EASM variability. (**A**) DG7 δ^18^O record (aquamarine) and sunspot number (orange; SILSO data, Royal Observatory of Belgium, Brussels). Black solid and dashed lines represent the mean and 2 SD of the δ^18^O record, respectively. Five brown triangles indicate five weak EASM events. SILSO, Sunspot Index and Long-term Solar Observations. (**B**) Quasi-11-year cycle in EASM (dark green) and sunspot number (orange) by band-pass filter. (**C**) Radionuclide-based reconstruction of changes in solar activity (*48*) (teal) and tree-ring reconstructed ENSO variance (*47*) (yellow). The yellow shadings highlight intervals of strong coupling between the EASM and solar activity during the strong 11-year solar cycle epoch (*8*).

Spectral analysis (fig. S7) and empirical mode decomposition (table S4A) reveal a notable decadal EASM variability. The ~10.3-year cycle, accounting for ~29.5% of the total variance throughout the past two centuries, is likely related to the 11-year solar cycle (Fig. 3A). Comparison of the EASM and sunspot number change using a band-pass filter (Fig. 3B) and a cross-wavelet transform (fig. S12) displays in-phase coherence during 1787–1870 and 1950–2007. Notably, decadal EASM changes exhibit a high correlation with solar forcing during the strong 11-year solar cycle epoch, with stronger EASM intensity positively correlated with higher solar irradiance (Fig. 3). A pronounced quasi-11-year cycle is also identified in simulated precipitation δ^18^O across the EASM region (*43*) and summer precipitation over northern China (*8*), as well as in observed summer precipitation pattern in China (1958–2020) (*9*), strongly corroborating the solar-EASM relationship. These observations support that solar activity played an important role in driving EASM changes through the top-down stratospheric response and/or the bottom-up tropical ocean-atmosphere response (*44*).

The phase asynchrony between the EASM and solar activity on the 11-year band is observed from 1870 to 1950 (Fig. 3), likely modulated by the enhanced internal climate variability. First, our analysis reveals a pronounced 2- to 4-year cycle in the EASM record from 1870 to 1950, which explains ~39.9% of the total variance (table S4B), largely related to the El Niño–Southern Oscillation (ENSO) variability. Second, the EASM record broadly resembles coral-based ENSO reconstruction from the central tropical Pacific (*45*, *46*). Sliding correlation analysis (29-year moving windows) further reveals strong EASM-ENSO coupling until the late 1950s, followed by weaker coupling thereafter (fig. S13). Third, the ENSO variance notably enhanced from 1870 to 1950 (*47*), coinciding with the interval of reduced solar irradiance (*48*) (Fig. 3C).

**Fig. 4.**
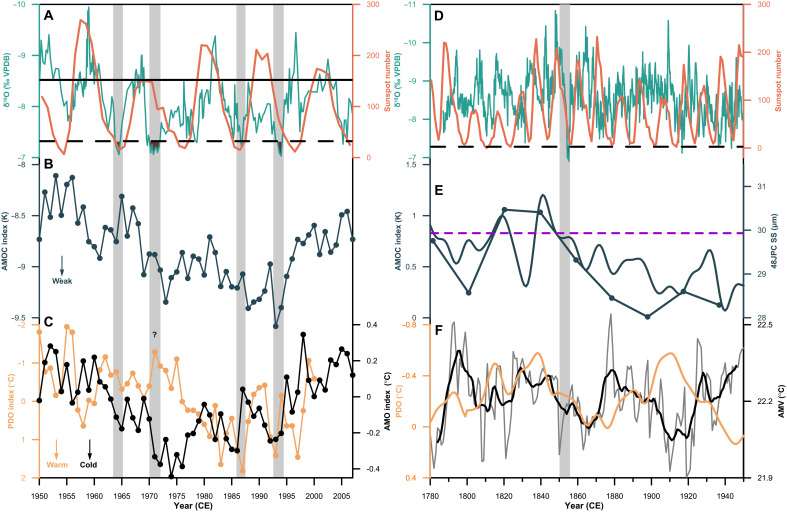
Weak EASM events and their dynamics. Left: Comparison between stalagmite-based EASM (aquamarine), sunspot number (**A**, orange; SILSO data, Royal Observatory of Belgium, Brussels), AMOC (*58*) (**B**, dark green; derived from HadISST data), PDO (*62*) (**C**, light orange; derived from UKMO Historical SST dataset for 1900–1981 and Reynold’s Optimally Interpolated SST (V1) for 1982–2001), and AMO (*64*) (C, black; derived from Kaplan SST data) records. Black solid and dashed lines represent the mean and 2 SD of the stalagmite δ^18^O record, respectively. Right: Same as the left panel, but comparison between proxy records. (**D**) Sunspot number. (**E**) The AMOC index (*59*) and mean sortable silt size (SS) record from the western North Atlantic (*60*) (dark green). The purple dashed lines represent the mean state of the AMOC intensity during the coldest period of the LIA (1400–1700 CE). (**F**) The PDO (*63*) (light orange) and Atlantic multidecadal variability (AMV) (*65*) (gray) records. The thick black line indicates a 9-year moving average of the AMV record. The vertical shadings indicate five weak EASM events, likely associated with reduced solar irradiance, weakened AMOC, cold AMO/AMV, and warm PDO phase. HadISST, Hadley Centre Global Sea Ice and Sea Surface Temperature; UKMO, U.K. Meteorological Office.

Furthermore, we identify five anomalously weak monsoon events over the past two centuries (Fig. 4), defined by the δ^18^O values exceeding 2 SD above the average (that is, > −7.31‰), following the approach used for Greenland ice core records (*49*). Four events occurred in the second half of the 20th century, around 1965, 1972, 1987, and 1994 (Fig. 4A), respectively, concurrent with widespread extreme droughts in northern China (*50*). The most pronounced one occurred in the 1850s (Fig. 4D), coinciding with a megadrought in northern China from historical documents (*51*). A recent tree-ring δ^18^O-based hydroclimate reconstruction from the Qinling-Bashan Mountains confirms a prominent dry period during 1850–1859 (*52*). This megadrought was also synchronous with megadroughts in India (*53*, *54*), southwestern North America (*55*), and West Africa (*56*) (fig. S14), collectively revealing a hemispheric-scale summer monsoon anomaly that underscores the interconnected nature of global climate dynamics during the LIA termination period.

Decadal-scale EASM variability is highly associated with the 11-year solar cycle (Fig. 4, A and D), and internal climate dynamics amplify and propagate these solar signals globally (*4*, *57*). The five weak monsoon events are associated with the rapid weakening in AMOC (Fig. 4, B and E) (*58*–*60*), even declining below mean levels observed during the coldest period of the LIA (1400–1700) (*59*, *60*). The AMOC weakening, combined with reduced solar irradiance, contributed substantially to abrupt EASM weakening through Northern Hemisphere cooling and a southward shift of ITCZ (*5*, *13*). Furthermore, the sea surface temperature (SST) pattern in the Pacific and Atlantic oceans can modulate the EASM circulation and intensify the north-south rainfall pattern in China (*4*, *8*–*10*, *61*). Here, four of the five weak monsoon events correspond to the low solar irradiance and warm PDO conditions (*62*, *63*) and all five correspond to the cold AMO/Atlantic multidecadal variability conditions (*64*, *65*) (Fig. 4, C and F). An exception is the weak monsoon event around 1972, which may be attributed to increased volcanic and anthropogenic aerosol emissions and the notable Northern Hemisphere cooling (fig. S11, B and C). Therefore, we confirm that solar forcing on decadal EASM variability can be amplified by Pacific and Atlantic SST patterns. The underlying dynamic mechanism involves the excitation of an anomalous SST pattern by high solar output, which resembles a cold PDO phase. The associated anomalous North Pacific anticyclone dominates the extratropical North Pacific and thereby enhances the EASM, which, in turn, results in abundant rainfall over northern China and deficient rainfall in southern China. In contrast, low solar output with a warm PDO phase can cause weak EASM intensity and drought in northern China (*8*–*10*). The Atlantic SST patterns further affect EASM via the global teleconnection pattern (*61*), enhancing the effect when the Pacific SST and Atlantic SST patterns are in opposite phases (*10*).

### Monsoon anomaly, population pressure, and social instability

**Fig. 5.**
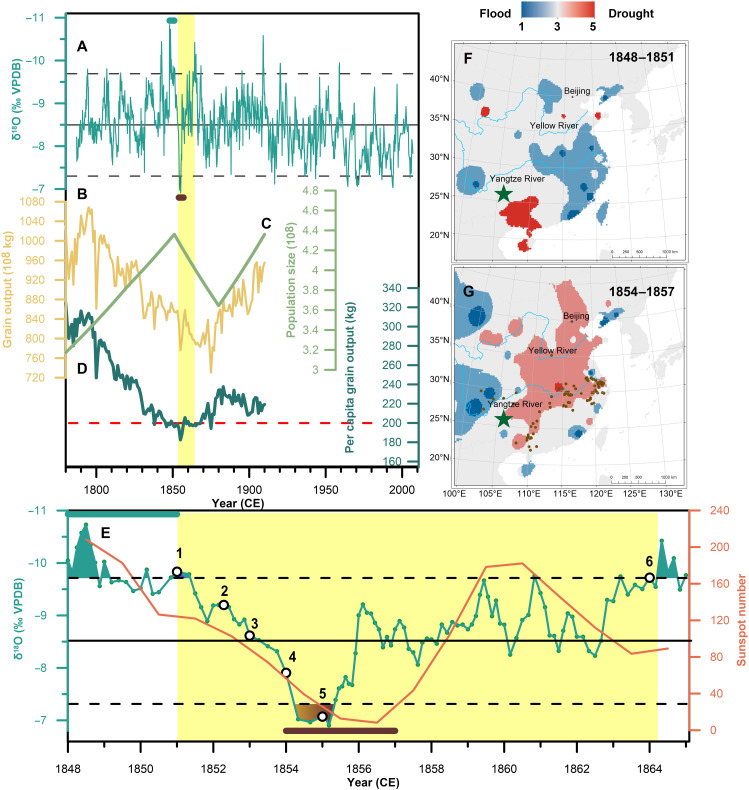
Decadal EASM anomalies contribute to the Taiping Rebellion. (**A**) The EASM record (aquamarine). The yellow vertical shading indicates the Taiping Rebellion (1851–1864). (**B** to **D**) Total national grain output (yellow), population size (pea green), and per capita grain output data (teal) (*67*). The red dashed line represents a threshold of extreme overpopulation, defined as per capita grain output of <200 kg at the national scale (*67*). (**E**) The EASM anomalies (aquamarine) and solar activities (orange) during the Taiping Rebellion (1, outbreak; 2, strategic transfer; 3, capital establishment; 4, grain crisis; and 5, Nian Rebellion). The aquamarine and brown horizontal bars in (A) and (E) indicate the strongest (1848–1851) and weakest (1854–1857) EASM intervals over the past 200 years, respectively. The spatial patterns of flood (**F**) and drought (**G**) during the strongest and weakest EASM intervals (*51*). The green star shows the location of Daoguan Cave. Brown dots in (G) indicate the outbreak location of major wars during the Taiping Rebellion (*72*).

Of note is the decadal-scale EASM anomaly that occurred during 1848–1859, marked by the most extreme δ^18^O excursion (~3.9‰ in amplitude) over the past 200 years (Fig. 5A). This EASM anomaly temporally coincided with the Taiping Rebellion (1851–1864), the most devastating civil conflict in modern Chinese history, accompanied by unprecedented casualties and demographic collapses (*16*, *17*, *66*, *67*). In the mid-19th century, the Qing government confronted severe financial stress due to continuous internal rebellions and foreign wars. Meanwhile, an acute societal crisis emerged, fueled by rapid population growth (~430 million in 1851; Fig. 5B) (*14*, *16*) alongside continuously decreasing grain production (Fig. 5C) (*16*, *67*). As the peasant majority subsisted on only 30 to 40% of the land, land annexation intensified their conflicts with the Qing government (*16*). The hydroclimate extremes in the 1850s caused widespread agricultural failures, which led to extreme overpopulation (*67*) (supplementary text 3 and Fig. 5D) and served as a critical trigger for the outbreak of the Taiping Rebellion.

The initial phase of this EASM anomaly featured an exceptionally strong EASM, evidenced by the δ^18^O minima (1848–1851; Fig. 5E), which induced large-scale flooding in the monsoon region of China (Fig. 5F), a pattern corroborated by the yearly charts of dryness/wetness (*51*). Historical documents show that more than 500 counties across six provinces in the Yellow River Basin were affected by flooding. The 1849 flood over the Yangtze River Basin was unprecedented in a century (*14*, *51*, *68*). This prolonged flood devastated farmland and displaced large numbers of peasants. Many refugees migrated to southwestern China in search of livelihoods, resulting in the precipitous decline of per capita arable land to <800 m^2^ in Guangxi Province by 1851 (*69*) (~1190 m^2^ on the national average level). Concurrently, droughts prevailed in southwestern China (Fig. 5F), evidenced by the DG7 trace element record and historical documents (fig. S15). As the drought intensified and spread across Guangxi, food shortages and price inflation exacerbated human-land conflicts and tensions between locals and migrant settlers (*70*, *71*). These compounded pressures became an important catalyst for the outbreak of the Taiping Rebellion in 1851 (*14*, *16*, *72*). Thereafter, food insecurity ultimately drove the Taiping forces’ invasion into the agricultural heartland in the middle and lower Yangtze River Basin in 1852, culminating in the establishment of Nanjing as their capital in 1853 (Fig. 5E) (*72*). Subsequently, the Taiping Heavenly Kingdom promulgated the Land System of the Heavenly Dynasty to address the conflict between population and land.

The second phase of this EASM anomaly is the anomalously weak monsoon event occurred in the 1850s, evidenced by the most positive δ^18^O values over the past 200 years (Fig. 5E). This prolonged EASM failure caused persistent and severe droughts (1854–1857) that affected from northern China to the Yangtze River Basin (Fig. 5G) (*51*). The resultant agricultural collapse with a sharp decline in grain production below subsistence levels (*67*) (<200 kg per capita; Fig. 5D) drove mass famine migrations that both swelled the Taiping ranks and triggered a catastrophic grain crisis by 1854 (*14*). Most war occurred in the Yangtze River Basin as contending forces vied for control of grain-producing regions (Fig. 5G). Meanwhile, the Nian Rebellion (1853–1868), a peasant uprising in northern China, formed a coalition in 1855, established a unified military structure, and coordinated operations to seize grain (*14*, *73*). The social unrest, crop failures, and fiscal crisis severely undermined the governing capacity of the Qing regime (*74*). The Qing government was compelled to mobilize regional militia forces to suppress the Taiping Rebellion. The Taiping Rebellion was eventually quelled in 1864 due to a variety of factors, including food shortages and internal strife, with an estimated death toll of 100 million (*14*, *66*).

Decadal-scale EASM anomalies, strongly modulated by solar activity and ocean-atmosphere processes in the Atlantic and Pacific oceans, trigger widespread floods and droughts across China. The marked flood-drought abrupt alteration from 1848 to 1857 (Fig. 5E) largely contributed to the outbreak and trajectory of the Taiping Rebellion, implicating that monsoon anomaly-induced harvest failures can fuel subsistence crises and spark social instability. Since the mid-20th century, anomalously weak monsoon events have increased in frequency (Fig. 4A). These climate challenges have been mitigated by government-led water resource management and food security policies, which alleviated disaster impacts and secured social stability. Therefore, our findings underscore that implementing sustainable water management and climate-smart agriculture is critical for addressing future climate extremes and achieving the Sustainable Development Goals.

## MATERIALS AND METHODS

### Sample DG7

Daoguan Cave, ~260 km west of Dongge Cave (*13*), is located on the eastern slopes of the Yunnan-Guizhou Plateau, southwestern China. The cave features a ~900-m-long gallery with two chambers and is overlain by ~20 m of Triassic limestone bedrock. The overlying flora is mainly composed of tussock scrub. One stalagmite (DG7) was collected from a poorly ventilated chamber with ~100% relative humidity. The mean annual temperature at the cave site is ~14°C. Local precipitation increases in early May as the EASM intensifies, marking the onset of the rainy season. The annual precipitation ranges from 800 to 1800 mm, with 76% occurring during the boreal summer (from May to September).

Stalagmite DG7, ~160 mm in length and 50 mm in diameter, was actively growing at the time of collection in 2007. When halved and polished, regular laminations can be observed in the depth section of 0 to 130.5 mm (figs. S2 and S3).

### ^230^Th and ^210^Pb dating methods

Two subsamples were collected for U-Th dating (table S1). The chemical procedures for U and Th separation and purification were similar to those described previously (*75*). The ^230^Th dating was performed at the Minnesota Isotope Laboratory with a multicollector inductively coupled plasma mass spectrometer (Thermo-Finnigan Neptune). For ^230^Th dating, correction for the effect of ^230^Th incorporated at the time of deposition is difficult for the young stalagmites. Any initial ^230^Th is always accompanied by a much larger amount of ^232^Th. Therefore, samples with ^230^Th/^232^Th ratios below an acceptable threshold were objectively identified and excluded (*76*, *77*). As shown in table S1, the ^230^Th/^232^Th ratios are too low (<10) for both dates. Therefore, the ^230^Th data have generally been regarded as unreliable (*76*, *77*). The ^230^Th dating results merely tell us that the DG7 stalagmite is extremely young.

Eleven subsamples were collected for ^210^Pb dating (table S2). About 0.12 to 0.30 g of powder of each sample were dissolved in concentrated HNO_3_ and spiked with ^209^Po. The Po was self-precipitated onto the silver plate in 0.1 N HCl solution under an 80°C water bath for 6 hours. The silver plate was counted for ^210^Po and ^209^Po by the ORTEC alpha spectrometer (*78*). The ^210^Pb dating was performed at the Institute of Karst Geology, Chinese Academy of Geological Science.

### XRD analyses

To confirm the mineralogy of the DG7 stalagmite, five subsamples (at the depths of 20, 60, 85, 100, and 120 mm) were drilled from the polished surface and analyzed for XRD (fig. S2). The measurements were performed on a Rigaku D/ Max 2500 x-ray diffractometer at Nanjing Normal University. The XRD results confirm that this stalagmite is entirely composed of calcite minerals.

### Stable isotope and trace element analyses

For stable isotope analyses (δ^18^O and δ^13^C), a total of 1516 subsamples were collected by knife shaving. The stable isotope analyses were performed on a Finnigan MAT-253 mass spectrometer in the Isotope Laboratory of Nanjing Normal University. The precision for δ^18^O is 0.06 and 0.03‰ for δ^13^C at the 1σ level. A total of 131 powdered samples were drilled with a carbide dental burr along the growth axis and used for trace element analyses at Chongqing Key Laboratory of Karst Environment, Southwest University. Each sample weighed 230 ± 50 μg and was dissolved in a solution of 3% HNO_3_ and 1% hydrofluoric acid (HF). Mg and Ca were analyzed using an inductively coupled plasma optical emission spectrometer (PerkinElmer), and Sr and Ba were measured using a single-collector inductively coupled plasma mass spectrometer (Element XR). International standard SLRS-5 was used to determine the accuracy and precision of the analyses. The precisions are better than 3% for Ca, 3% for Mg, 5% for Ba, and 10% for Sr.

### Model simulations

The CESM-LME (*26*) is the largest available set of single model simulations spanning the past millennium, thereby providing a unique testbed for exploring climate variability across multiple timescales. Here, we focus on the “All Forcing” ensemble (*n* = 13), forced by solar variability, volcanic eruptions, land-use, greenhouse gas, ozone-aerosol, and orbital changes (*26*). In addition, we also used the isotope-enabled Last Millennium simulations conducted by the iCESM 1.2 (*27*), with external forcings corresponding to the configuration of nonisotopic CESM-LME. The resolution of the model for the atmosphere/land components is ∼2°, and the resolution for the ocean/sea-ice components is ∼1°. The iCESM-LME simulations include a preindustrial control experiment, three all-forcing experiments, and five single-forcing sensitivity experiments, including two volcanic eruption experiments, one total solar irradiation experiment, greenhouse gas experiment, and orbital parameter experiment.

## Supplementary Materials

### This PDF file includes:

Supplementary Texts 1 to 3 Figs. S1 to S17 Tables S1 to S5 References
