# Supplementary material for: Decadal East Asian monsoon anomalies and implications for societal conflicts
Source: Sci Adv. 2026 May 6;12(19):eaee1648. doi: 10.1126/sciadv.aee1648 (PMC13148317; doi:10.1126/sciadv.aee1648)
Supplement: Supplementary file 1 — Supplementary Texts 1 to 3 Figs. S1 to S17 Tables S1 to S5 References [file sciadv.aee1648_sm.pdf]

Science Advances
AAAS

Supplementary Materials for

Decadal East Asian monsoon anomalies and implications for societal conflicts

Kan Zhao *et al.*

Corresponding author: Changchun Huang, huangchangchun@njnu.edu.cn

Sci. Adv. **12**, eaee1648 (2026)
DOI: 10.1126/sciadv.aee1648

This PDF file includes:

Supplementary Texts 1 to 3
Figs. S1 to S17
Tables S1 to S5
References

Supplementary Text

Text 1. $\delta^{13}C$ record and vegetation evolution

The $\delta^{13}C$ values vary from -8.74‰ to -4.02‰, with an average value of -7.00‰. The $\delta^{13}C$ record displays distinct annual cycles, with amplitudes larger than ~1‰ on average (Fig. 1B and Fig. S3C). In principle, speleothem $\delta^{13}C$ values are linked to vegetation (e.g., vegetation type and density) because cave drip waters first pass through the overlying soil, and soil organic matter is derived from vegetation (*79, 80*). Numerous field investigations and cave monitoring studies confirm that the dominant vegetation is the C_3 type in Guizhou province, with average $\delta^{13}C$ values ranging from -32‰ to -25‰. Consequently, the average $\delta^{13}C$ values in drip waters and their corresponding speleothems range from -13.2‰ to -8.6‰ and -11.1‰ to -6.2‰, respectively (*81–84*). The latter is approximately equal to the summer $\delta^{13}C$ values (-8.7‰ – -5.9‰) in stalagmite DG7, indicating that the DG7 $\delta^{13}C$ values primarily inherit the $\delta^{13}C$ signal of C_3 vegetation changes. Therefore, we interpret the DG7 $\delta^{13}C$ record as a proxy of vegetation changes, with reduced vegetation density and soil microbial activity leading to reduced soil CO_2 production and high speleothem $\delta^{13}C$ values.

The most significant characteristic is the two distinct positive excursions in $\delta^{13}C$ values centered in 1945 and 1958 A.D., respectively, with amplitudes of ~4.4‰ (Fig. S5). We interpret the two $\delta^{13}C$ anomalies as the results of human-induced deforestation, likely related to China's War of Resistance against Japanese Aggression during World War II (1937–1945) and Great Leap Forward (1958–1960), respectively. The former event (forest cover declined from ~3.69 million hm^2 in 1900 to ~2.11 million hm^2 in 1949 (*85*)) is largely attributed to the rapid population growth (~113,000 people per year; Fig. S5) and the intensified industrial and agricultural development in Guizhou Province during the wartime period (*86*). The latter may be due to large-scale deforestation during the Great Leap Forward, when almost all trees (except a few forest reserves) were stripped to make charcoal for steel production across China (*85*). The steel production in Guizhou Province and China increased significantly from 1958 to 1960, and then sharply decreased in 1962 (Fig. S5).

Additional evidence supporting the anthropogenic effects on the two vegetation destructions is provided by sedimentary records from nearby plateau lakes. As shown in Fig. S6A, variations in char/soot ratio from Fuxian Lake (*23*) are broadly similar to DG7 $\delta^{13}C$ changes, within dating uncertainties of both records. This char/soot record indicates two peaks of the anthropogenic biomass burning during the interval of 1940–1960 A.D. Sedimentary record of charcoal accumulation rates in Xingyun Lake (close to Fuxian Lake) (*22*) also demonstrates two notable biomass burning peaks during 1940–1960 and matches well with DG7 $\delta^{13}C$ record (Fig. S6B). Furthermore, one or two biomass burning peaks during 1940–1960, revealed by the char/soot index (Fig. S6C-E) and $\delta^{13}C$ of soot (Fig. S6c-e) from Dianchi Lake, Erhai Lake, and Dazecuo Lake, are likely related to the vegetation destruction in the mid-20th century (*87*).

Text 2. Trace elements and regional hydrology reconstruction

The Mg/Ca, Sr/Ca, and Ba/Ca profiles, with a temporal resolution of ~1.6 years on average, show patterns broadly similar to the $\delta^{13}C$ record over the last 200 years (Fig. S2 and Table S5). Despite a series of complex processes, such as prior calcite precipitation (PCP), incongruent calcite dissolution, water-rock interaction, aerosol input and selective leaching, have potential control over the incorporation of trace elements into speleothems, significant correlations between Mg/Ca, Sr/Ca and Ba/Ca records from stalagmite DG7 (Table S5) can be interpreted as the results of PCP (*88,89*). The PCP effect is further confirmed using the mathematical model

(*90*) (Fig. S16). These observations are in good agreement with previous multi-proxy studies of short-term (*21,89*) (seasonal to annual) and long-term (*91, 92*) (centennial to orbital) hydroclimate changes.
Here, we performed Principal Component Analysis (PCA) to extract the leading mode of the Mg/Ca, Sr/Ca, and Ba/Ca records (from here referred to as "TE_{PC1}"). The TE_{PC1}, accounting for ~68% of the total variance, represents the dominant regional hydrologic signals embedded in these records (Fig. S10). Correlation between the TE_{PC1} record and instrumental rainfall data supports this interpretation, with an increase in TE_{PC1} value corresponding to a decrease in annual rainfall amount (Fig. S17).

Text 3. Estimation of population pressure and its thresholds

According to the theoretical framework from ref. (*67*), the annual fluctuation of human carrying capacity and per capita grain output in China from 1730 to 1910 are estimated using the following equations:

Human carrying capacity = Total cultivated land area × Max yield per land unit × Harvest index

Per capita grain output = Human carrying capacity / Total population size

During the Ming and Qing dynasties, the average per capita grain output in China fluctuated between 250 and 300 kg. The period is regarded as "normal" when per capita grain output is >250 kg, "overpopulated" when per capita grain output is between 200 and 250 kg, and "extremely overpopulated" when per capita grain output is <200 kg (67).

Fig. S1.

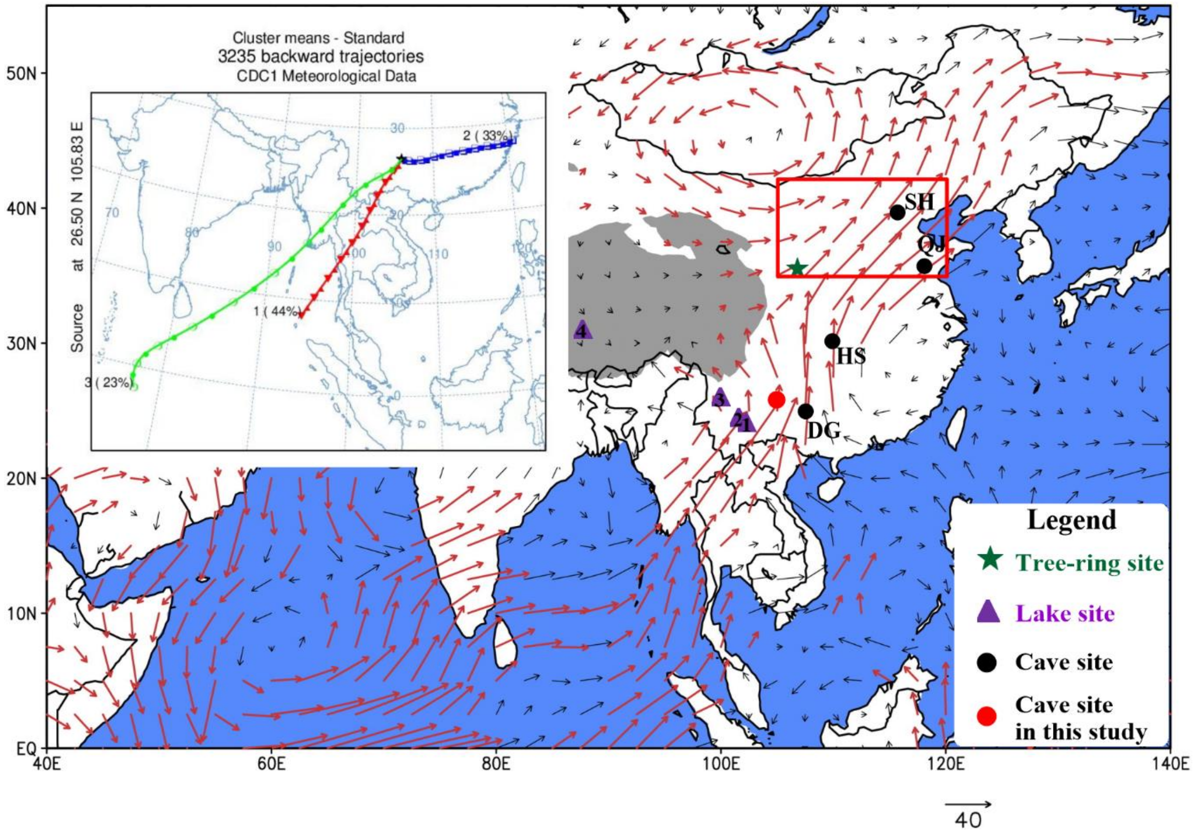

Fig. S1. Study sites and summer moisture transports in eastern China. Composite difference of moisture transport (units: kg m^{-1} s^{-1}, vertically integrated from the surface up to 300 hPa) in summer between strong (1969–1984) and weak (1985–2006) Indian summer monsoon (ISM) years based on NCEP/NCAR reanalysis data (*93*). Red vectors indicate a dominant moisture transport from the Indian Ocean (significant at the 95% confidence level). The figure is modified from ref. (*94*). The inset map shows the multi-year (1990–1999) mean air mass backward trajectories retrieved for summer (May-September) at the study site, confirming ~67% moisture transport from the Indian Ocean (*95*). Cave sites mentioned in the text include Daoguan (this study), Dongge (DG) (*32*), Heshang (*HS*) (*33*), Qujia (*QJ*) (*7*) and Shihua (SH) (*6*) caves. These cave sites are located on the moisture transport pathway from the Indian Ocean. Purple triangles indicate lake locations, including Lake Fuxian (*23*) and Xingyun (*22*) (NO.1), Lake Dianchi (NO.2), Lake Erhai (NO.3), and Lake Dazecuo (NO.4). The green star shows the tree-ring sampling site (*39*). The red box marks the region of northern China (105°E to 120°E, 35°N to 42°N).

Fig. S2.

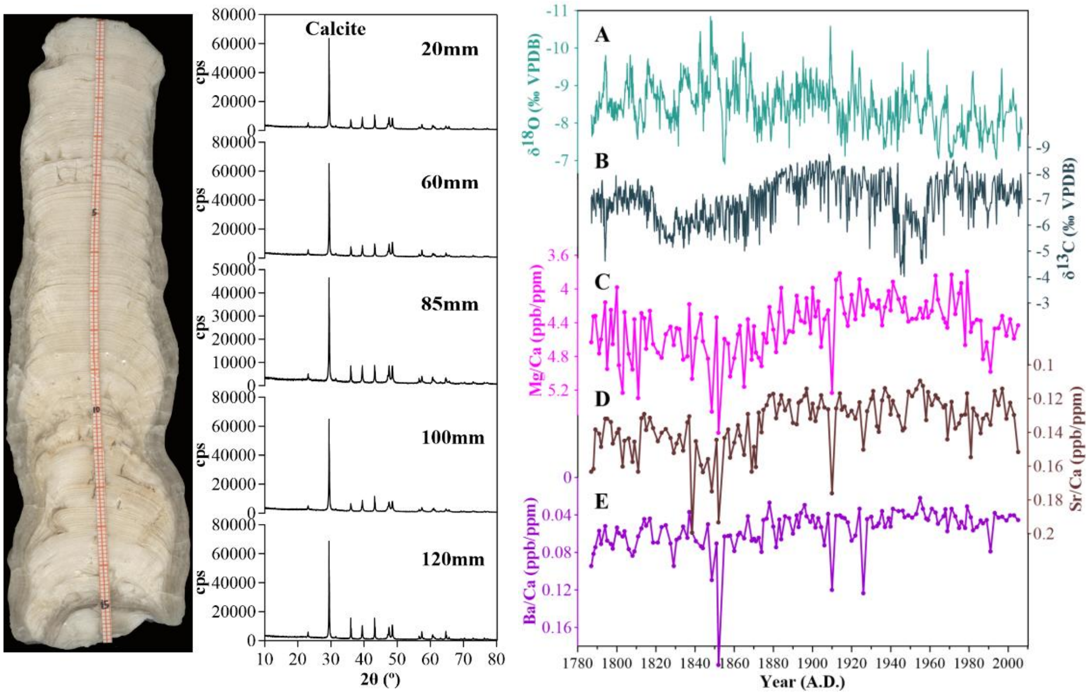

Fig. S2. Polished section (left), X-ray diffraction (XRD) analysis results (middle) and multi-proxy data (right) of stalagmite DG7. The XRD results confirm that this stalagmite is entirely composed of calcite minerals. Right panel: (A) $\delta^{18}O$ (aquamarine), (B) $\delta^{13}C$ (dark green), (C) Mg/Ca (magenta), (D) Sr/Ca (brown), and (E) Ba/Ca (purple).

Fig. S3.

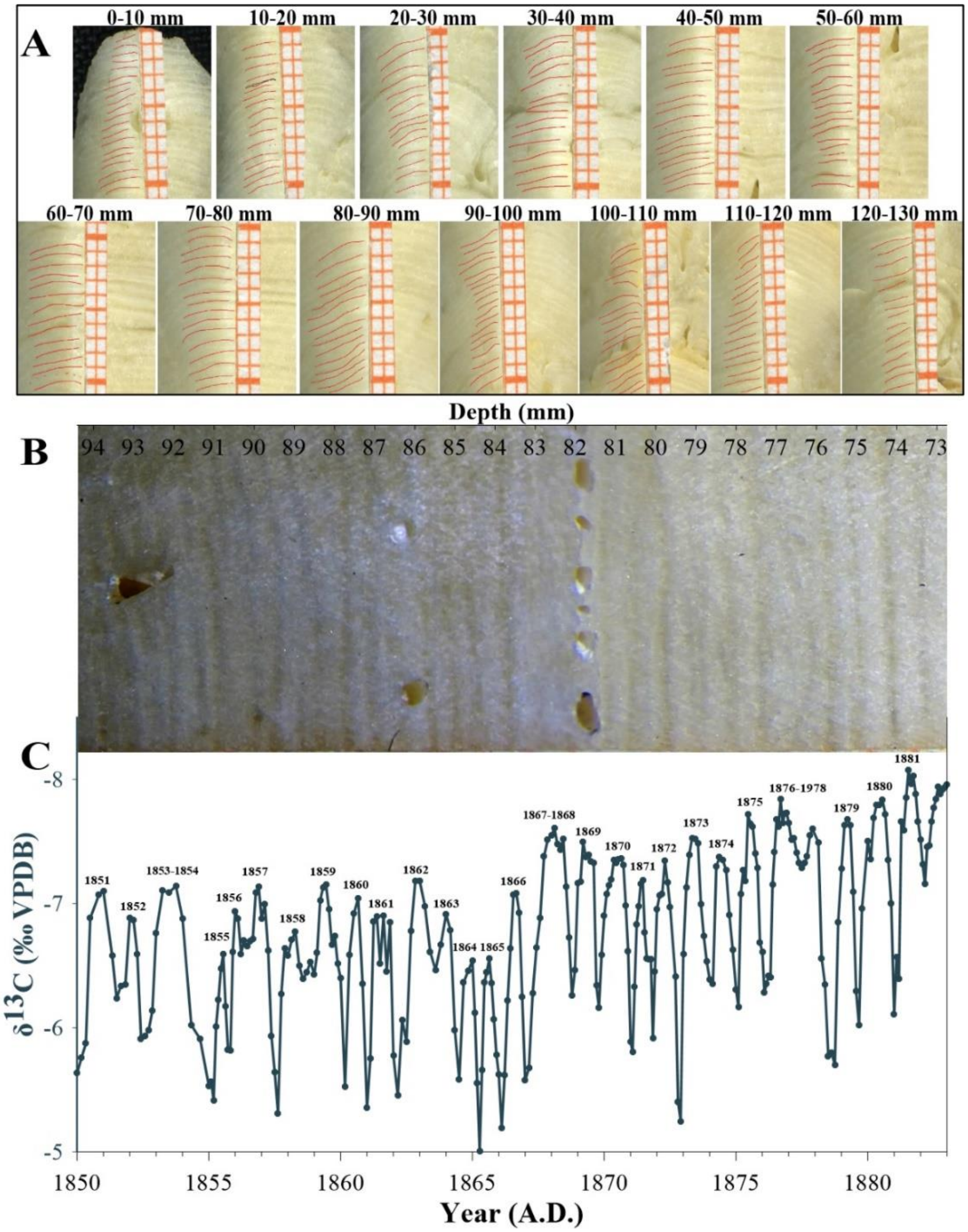

Fig. S3. Annual layers and annual $\delta^{13}C$ cycles in DG7. A total of 220 annual layers were counted to a depth of ~130.5 mm (A), with an accumulative error of 4 years (multiple counts by different people). The annual layers consist of couplets of white-porous and dark-compact calcites (B), which correspond to summer (wet season) and winter (dry season) formation, respectively (*96*). The $\delta^{13}C$ values are primarily controlled by regional vegetation changes (Supplementary Text 1). Relatively positive $\delta^{13}C$ values reflect reduced vegetation density in winter, whereas more negative values reflect increased vegetation in summer (C). Therefore, relatively positive $\delta^{13}C$ values are associated with the dark laminae and more negative values with the white laminae (B and C). The clear annual layers and seasonal $\delta^{13}C$ changes further confirm that the stalagmite DG7 grew continuously without hiatus.

Fig. S4.

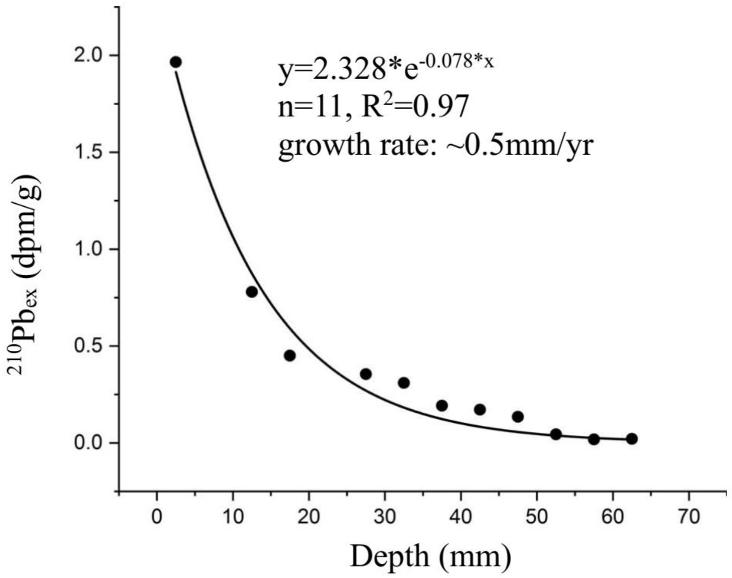

Fig. S4. The excess ^{210}Pb profile. The ^{210}Pb dating results indicate an exponential decay of excess ^{210}Pb. Based on the fitting of excess ^{210}Pb decay trend, an average growth rate of ~0.50 mm/year is calculated for the upper ~60 mm for the stalagmite. Consequently, carbonate deposits above the depth of ~60 mm should be younger than 110 yr before the present.

Fig. S5.

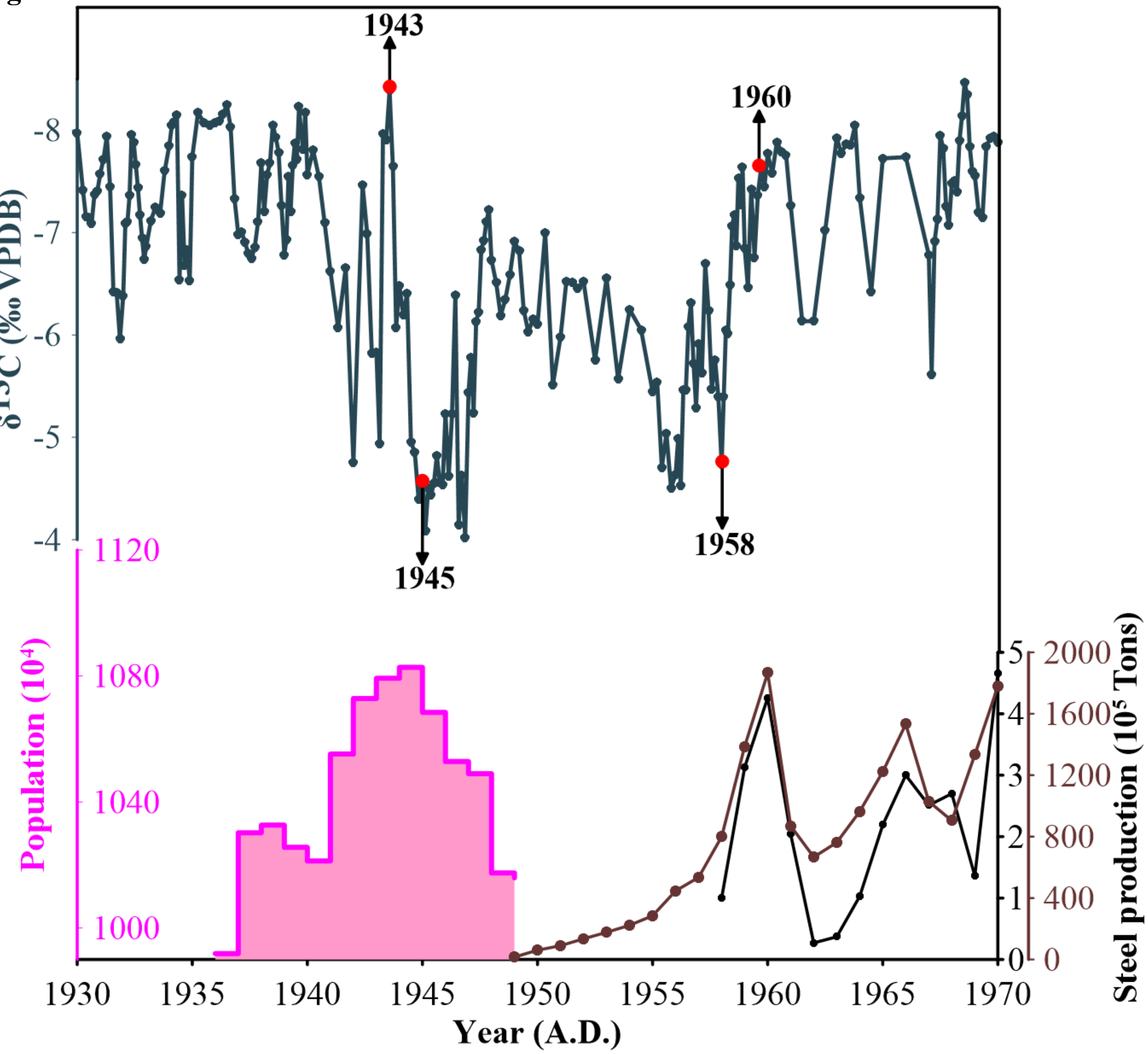

Fig. S5. Human-induced large-scale deforestation. The δ^{13}C anomalies and possible anthropogenic effects (population changes in Guizhou province (*86*), pink bars; steel productions in Guizhou and China, black and dark brown curve).

Fig. S6.

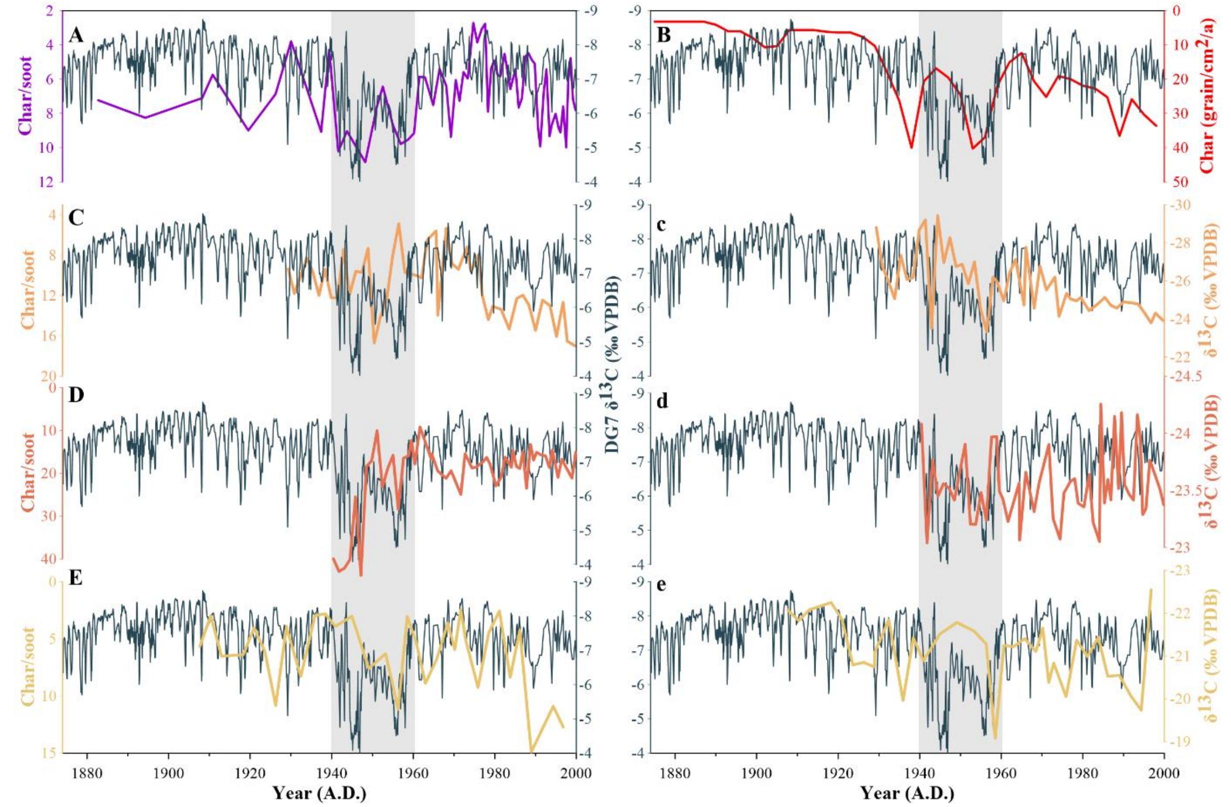

Fig. S6. Comparison between stalagmite and lake records. (A and B) Sedimentary records of char/soot ratio (purple) from Fuxian Lake (*23*) (24º21′N – 24º37′N, 102º49′E – 102º57′E) and charcoal accumulation rates (red) in Xingyun Lake (*22*). Sedimentary records of char/soot ratio and δ^{13}C of soot from Dianchi Lake (C and c, light orange, 24º40′N – 25º02′N, 102º36′E – 102º47′E), Erhai Lake (D and d, orange, 25º25′N – 26º16′N, 99º32′E – 100º17′E) and Dazecuo Lake (E and e, yellow, 31º49′N – 31º59′N, 87º25′E – 87º39′E) (*87*). Stalagmite DG7 δ^{13}C record is the dark green line. The vertical bar indicates two anomalies in δ^{13}C values centered in 1945 and 1958 A.D.

Fig. S7.

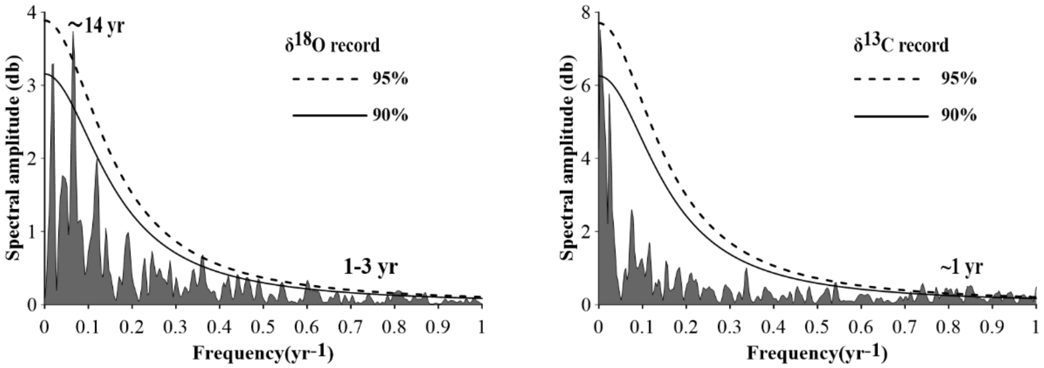

Fig. S7. Power spectrum analysis of DG7 $\delta^{18}O$ and $\delta^{13}C$ records.

Fig. S8.

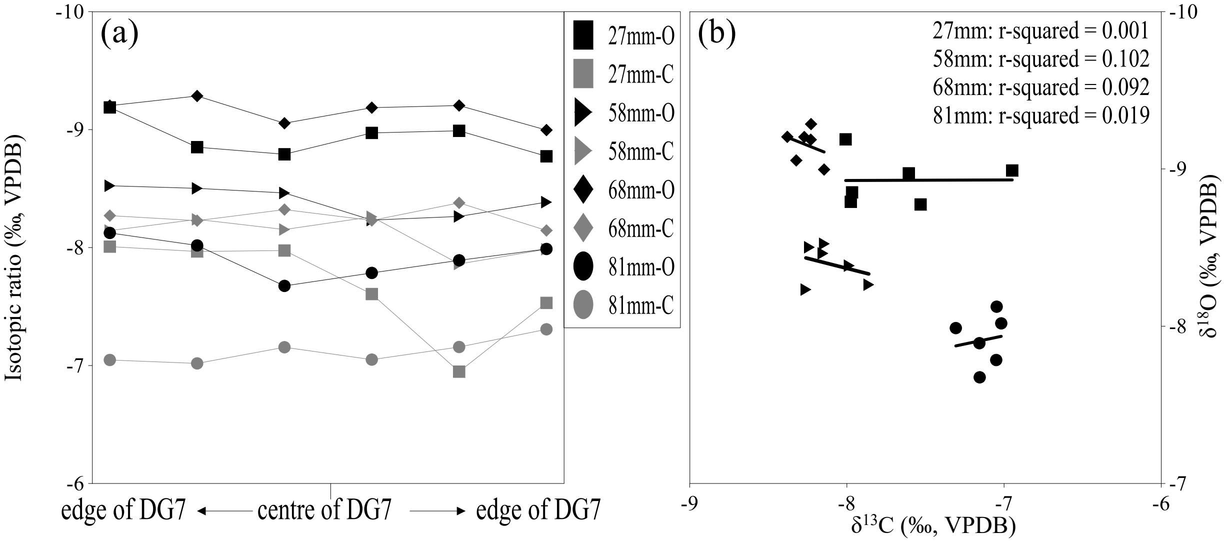

Fig. S8. The Hendy Test (*24*). The Hendy Test was performed on four individual growth laminae of sample DG7 (at the depths of 27 mm, 58 mm, 68 mm, and 81 mm). The results show that most $\delta^{18}O$ and $\delta^{13}C$ variations along the same layer are less than 0.3‰ and 0.4‰, respectively, and correlations between them are statistically insignificant, suggesting that the calcite was deposited close to equilibrium.

Fig. S9.

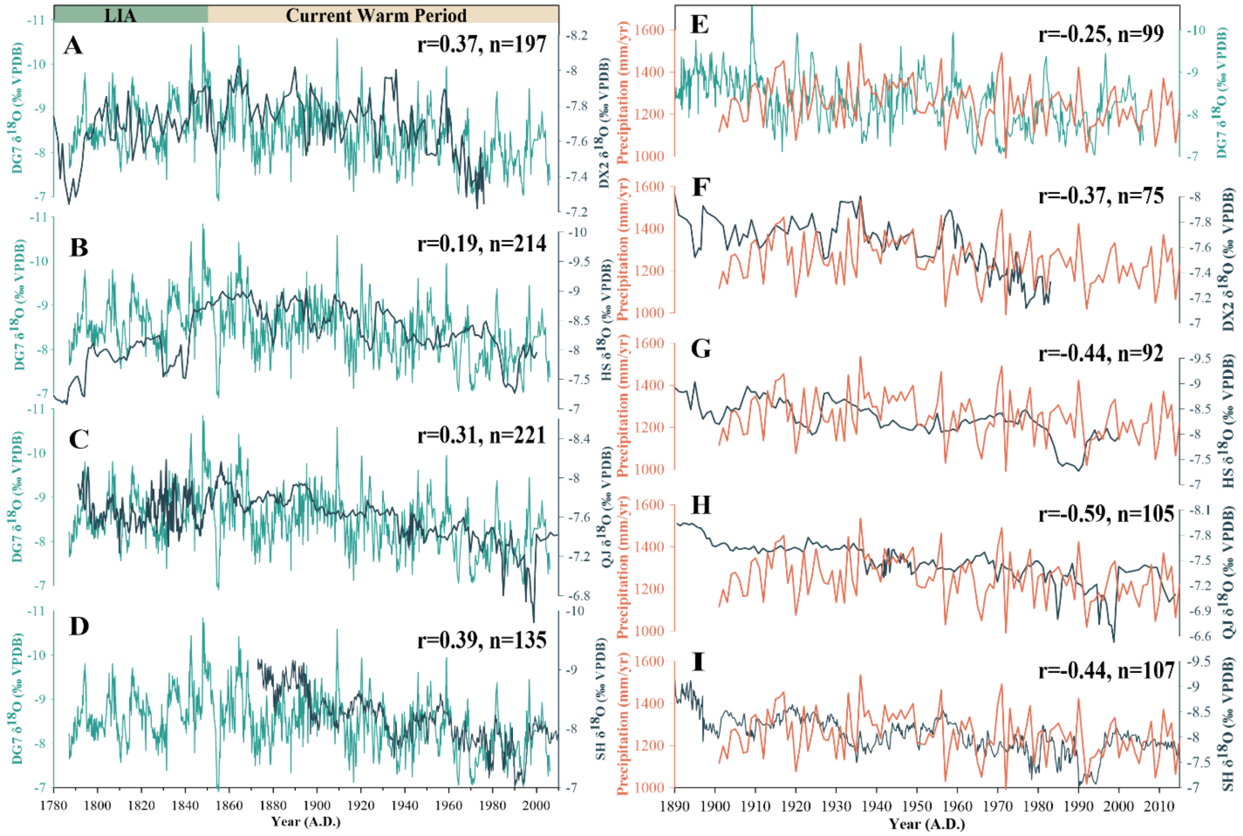

Fig. S9. Comparison between stalagmite $\delta^{18}O$ records and ISM rainfall. Left panel: Comparison of stalagmite $\delta^{18}O$ records from southwestern (A, Dongge (*32*)), central (B, Heshang (*33*)) and northern China (C, Qujia (*7*) and D, Shihua (*6*)) along the moisture transport pathway from the Indian Ocean (shown in Fig. S1). DG7 record is the aquamarine line. All data are linearly interpolated to produce a uniform 1-year temporal resolution and then calculated correlation coefficient (*97*). Right panel: Correlation between observed ISM rainfall (*40*) (orange line) and stalagmite $\delta^{18}O$ records from Daoguan (E), Dongge (F), Heshang (G), Qujia (H), and Shihua (I) caves. All data are linearly interpolated to produce a uniform 1-year temporal resolution and smoothed with a 9-year moving average, and then calculated correlation coefficient (Significant at the $p < 0.01$ level).

Fig. S10.

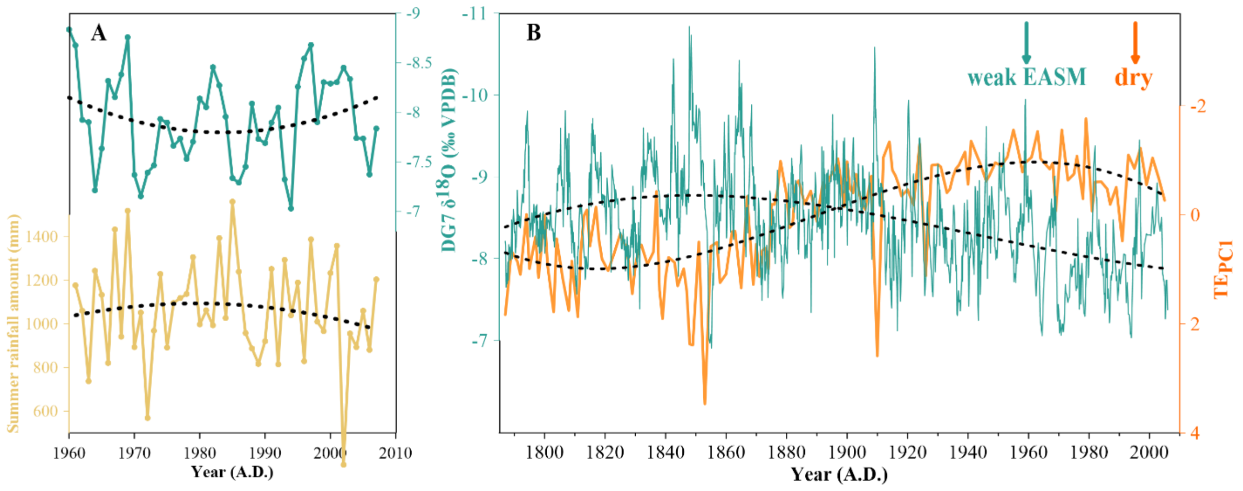

Fig. S10. Relationships between EASM intensity and regional hydroclimate variations. The EASM variations derived from $\delta^{18}O$ record (aquamarine) are negatively related to the regional hydrological changes from instrumental data (yellow, A) and trace element records (orange, B). The instrumental summer rainfall amount data (A) is from the Puan meteorological observatory, ~20 km to the northwest of Daoguan Cave. The black dashed lines indicate a 2nd order polynomial fitting for these records.

Fig. S11.

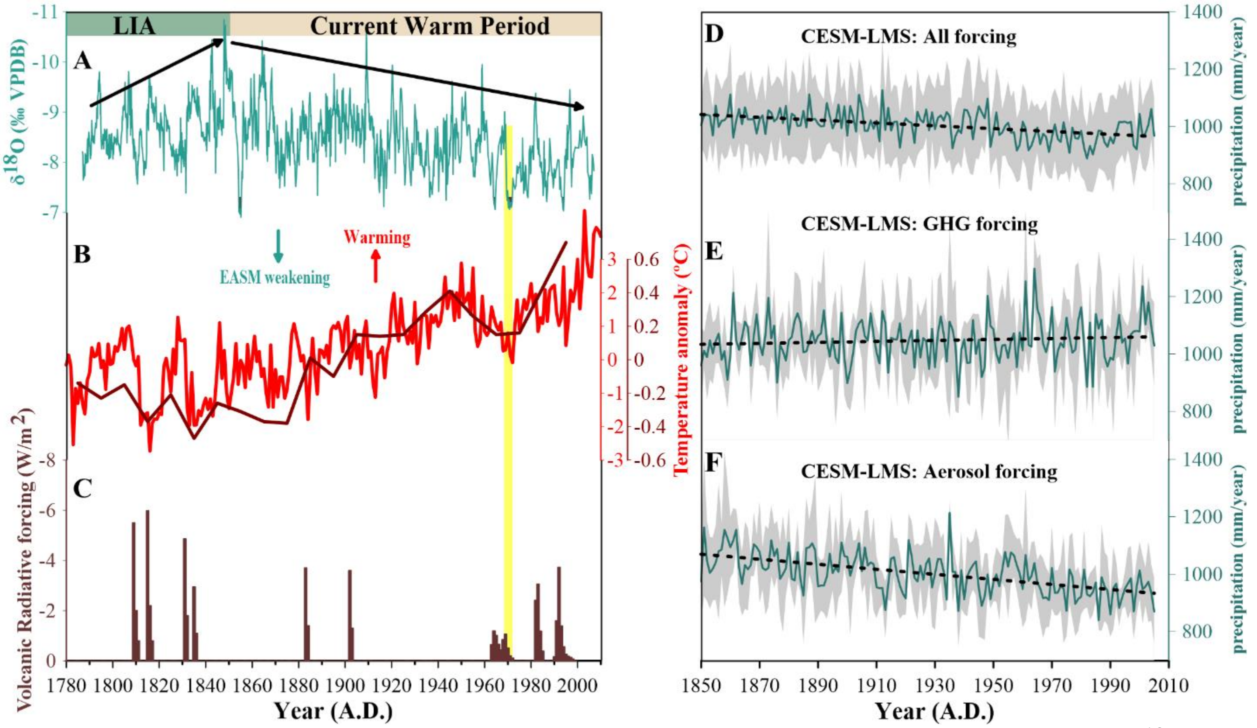

Fig. S11. Aerosol forcing on the EASM weakening since 1850s. Left panel: (A) DG7 $\delta^{18}O$ record (aquamarine). (B) Temperature anomalies (Z-score) relative to the period of AD 1750-1950 in Eurasian (red) (*98*) and China (dark red) (*99*). (C) Tropical Volcanic Radiative Forcing (brown) (*100*). The EASM enhanced until ~1850s and then gradually weakened (black arrows). The yellow vertical shading indicates the weak monsoon event around 1972, which may be attributed to increased volcanic and anthropogenic aerosol emissions and the notable Northern Hemisphere cooling. Right panel: Annual precipitation in northern China (35°N to 42°N, 105°E to 120°E) in CESM-LME all forcing simulation (D), Greenhouse Gas forcing simulation (E) and aerosol forcing simulation (F) (*26*). The teal line represents the mean, the shadow area represents mean ± 1 standard deviation, and the black dash line represents the long-term trend.

Fig. S12.

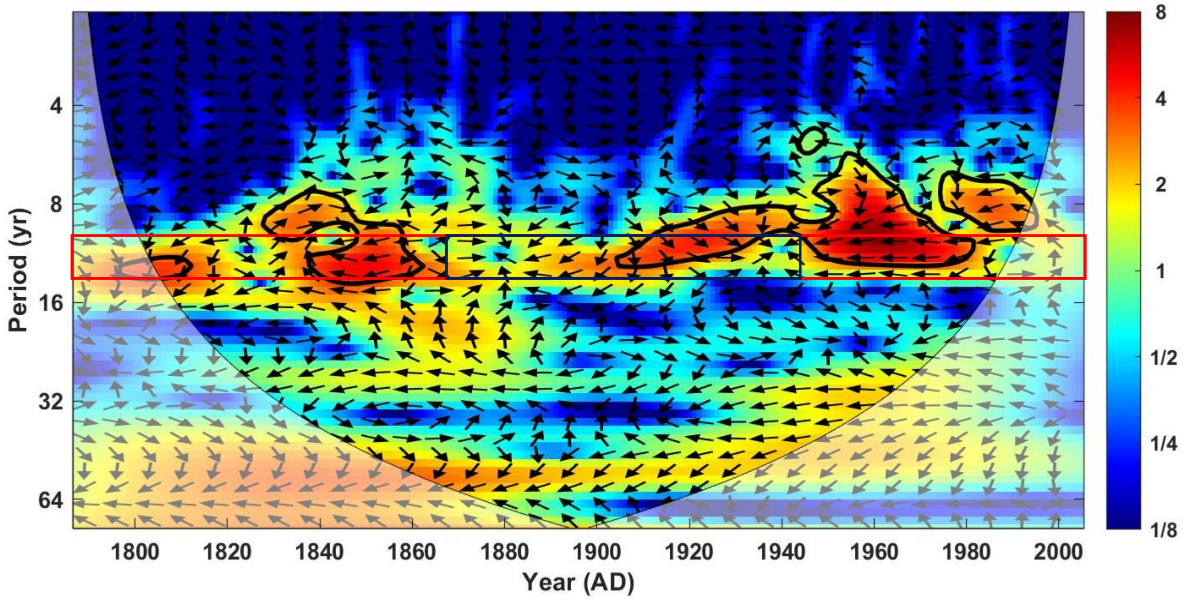

Fig. S12. Cross wavelet analysis of sunspot number (SILSO data, Royal Observatory of Belgium, Brussels) and DG7 $\delta^{18}O$ record. The Y-axis represents changes in coherence in the time domain, and the X-axis indicates changes in coherence on continuous timescales. The black arrows (right/left) indicate the phase relationship (in-/anti-phased) of the two records. The red boxes outline the in-phase relationship between EASM and solar activity on ~11-year cycle. The blue box indicates the out-of-phase relationship of the two records on ~11-year cycle during 1870-1950 A.D.

Fig. S13.

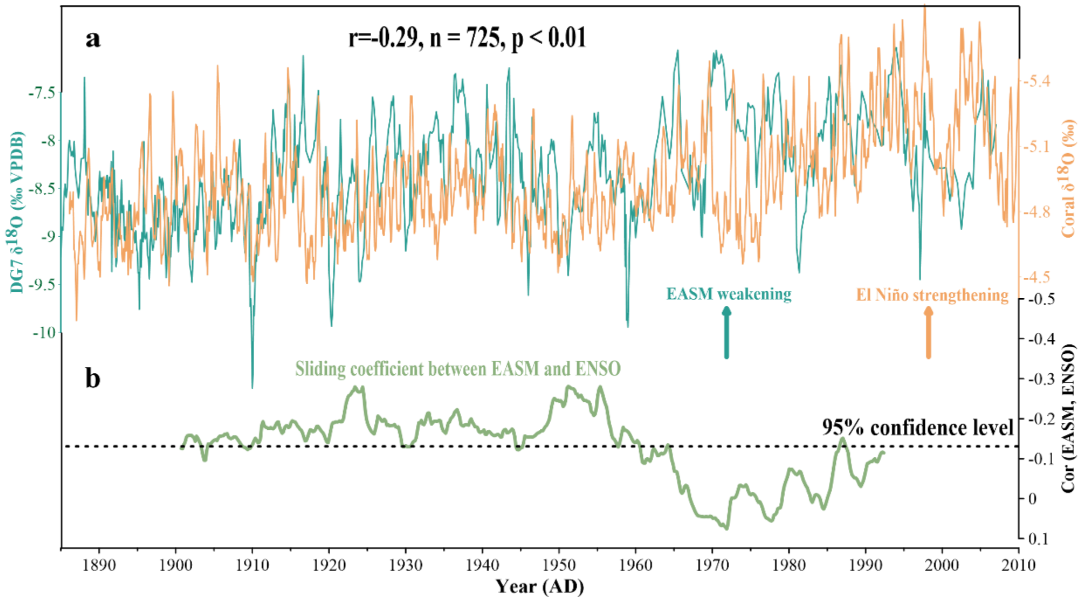

Fig. S13. Comparison between EASM and ENSO. (a) Relationship between DG7 $\delta^{18}O$ (aquamarine) and modern coral-based ENSO record (*45, 46*) (light orange). The Y axis for the DG7 record is oriented to show a weak EASM with El Niño conditions. The correlation coefficient is calculated with a 2-month equal interval interpolation. (b) The 29-year sliding coefficient between EASM and ENSO (pea green). The dashed line denotes the 95% confidence level.

Fig. S14.

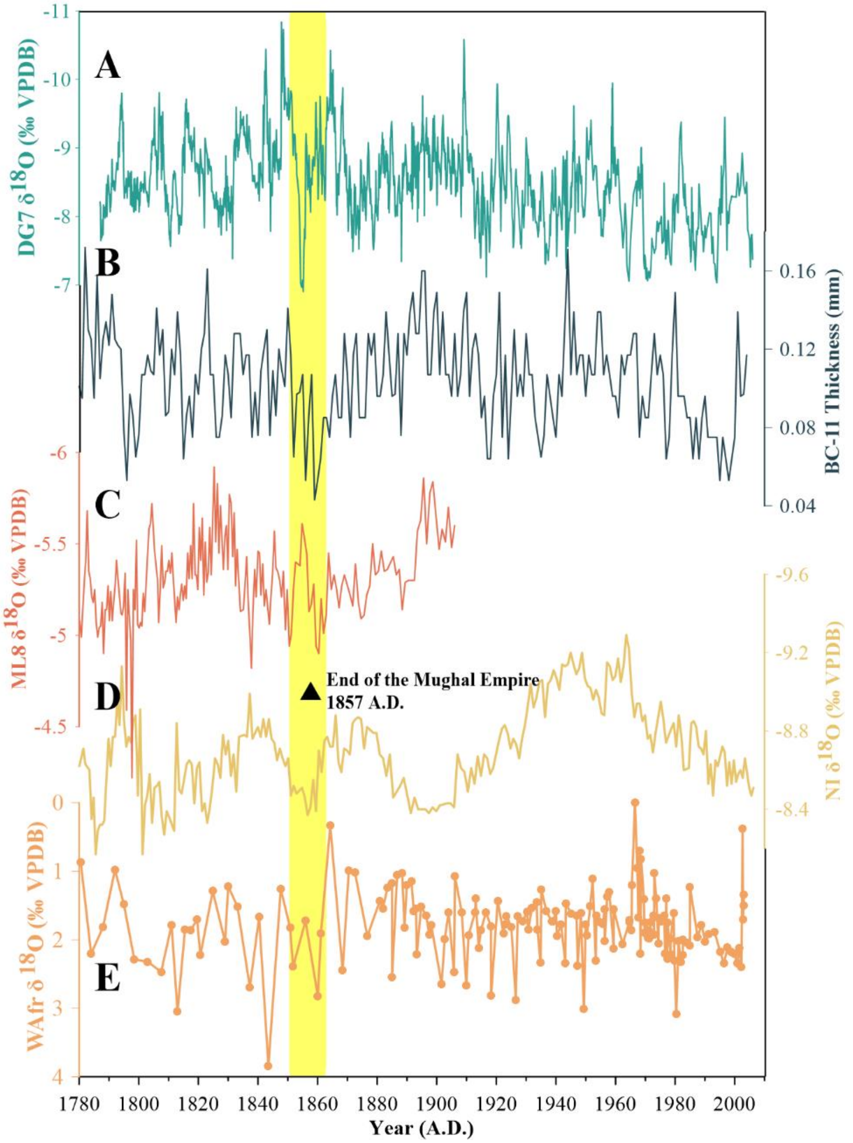

Fig. S14. One hemispheric scale megadrought around the 1850s. Comparison between stalagmite DG7 $\delta^{18}O$ (A), annual band thickness data from southwestern North America (*55*) (B), $\delta^{18}O$ records from Mawmluh Cave (*54*), northeast India (C), and Sahiya Cave (*53*), northern India (D), and lake sediment $\delta^{18}O$ data from West Africa (*56*) (E). All records indicate one megadrought in 1850s (yellow vertical shading), which likely contributes to the Taiping Rebellion (1851 – 1864 A.D.) in China and the end of the Mughal Empire (1857 A.D., black triangle) in India (*54*). Based on the precise layer-counting age model, the EASM intensity (DG7 $\delta^{18}O$, A) is broadly similar to the moisture amount (*55*) (annual band thickness, B) in southwestern North America, with a correlation coefficient of -0.29 (n=212, 7-year running mean).

Fig. S15.

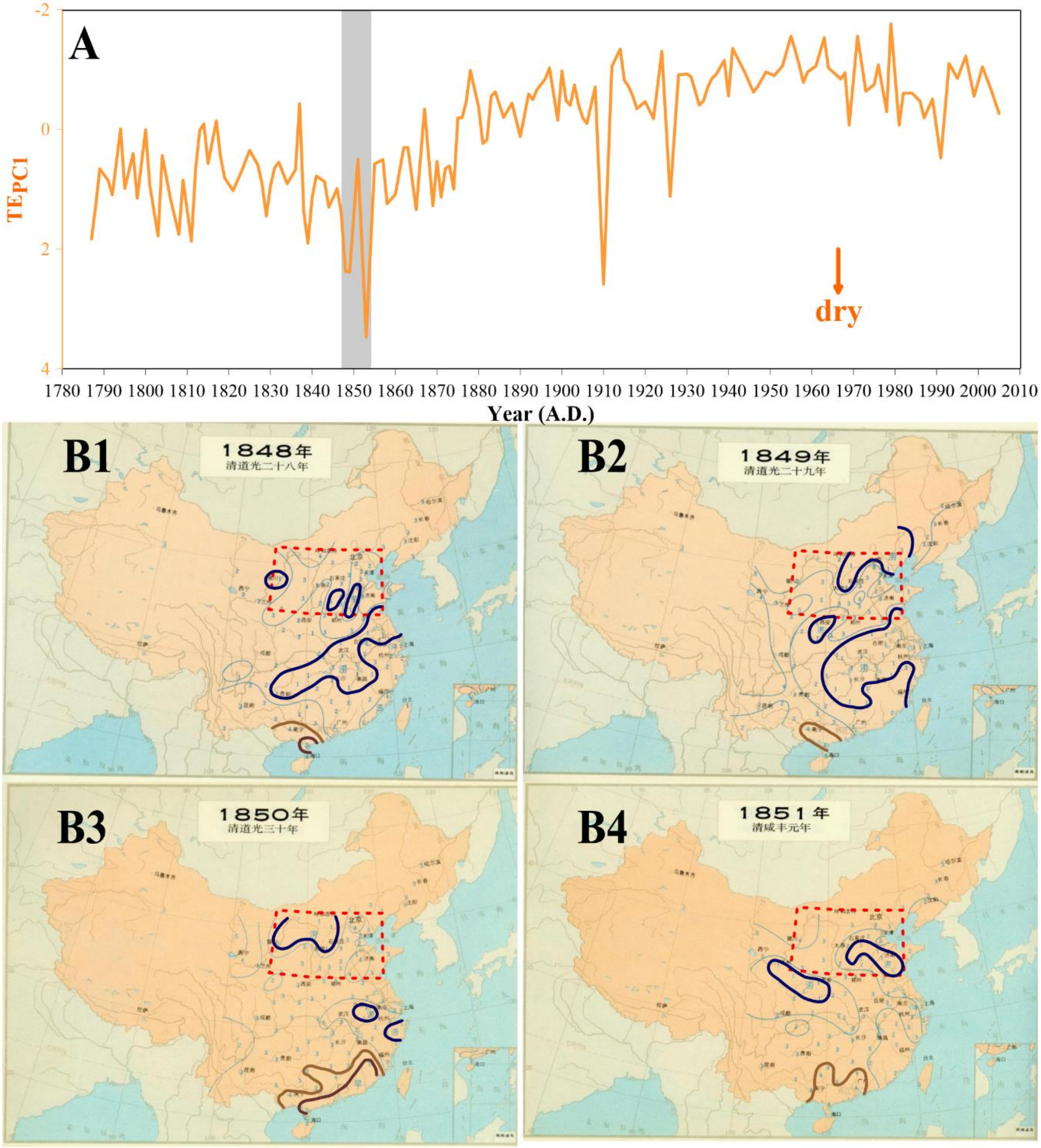

Fig. S15. Floods and droughts in monsoon regions of China from AD 1848 – 1851. (A) The hydrological changes in southwestern China from TE_{PC1} record (orange). The vertical shading indicates droughts around 1847-1854. **(B1–B4)** Average flood/drought conditions sourced from the yearly charts of dryness/wetness in China (*51*). Deep blue lines highlight the regions of floods. Brown and dark brown lines indicate the regions of droughts and severe droughts. The red box marks the region of northern China (105°E – 120°E, 35°N – 42°N).

Fig. S16.

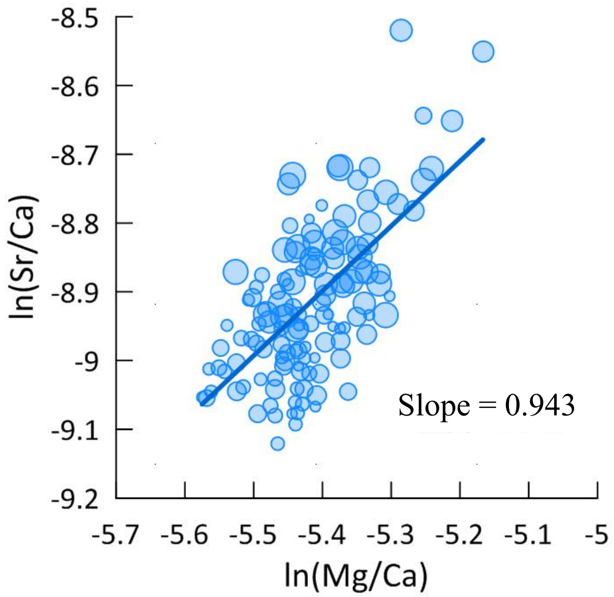

Fig. S16. The prior calcite precipitation (PCP) Test using the mathematical model (*90*). If PCP modulates Mg/Ca and Sr/Ca variabilities, the theoretical slope of ln(Sr/Ca) vs. ln(Mg/Ca) could be given by (Kd_{Sr} - 1) vs. (Kd_{Mg} - 1). Assuming a mean annual cave temperature of 14°C at the Daoguan site (approximately equal to the mean annual temperature outside), the Kd_{Sr} is 0.1 (*101*) and Kd_{Mg} is 0.015-0.025 (*102*), and then the slope should be around 0.92. In DG7, the linear slope of ln(Sr/Ca) vs. ln(Mg/Ca) is higher (0.943) than the expected gradient, indicating a strong PCP effect.

Fig. S17.

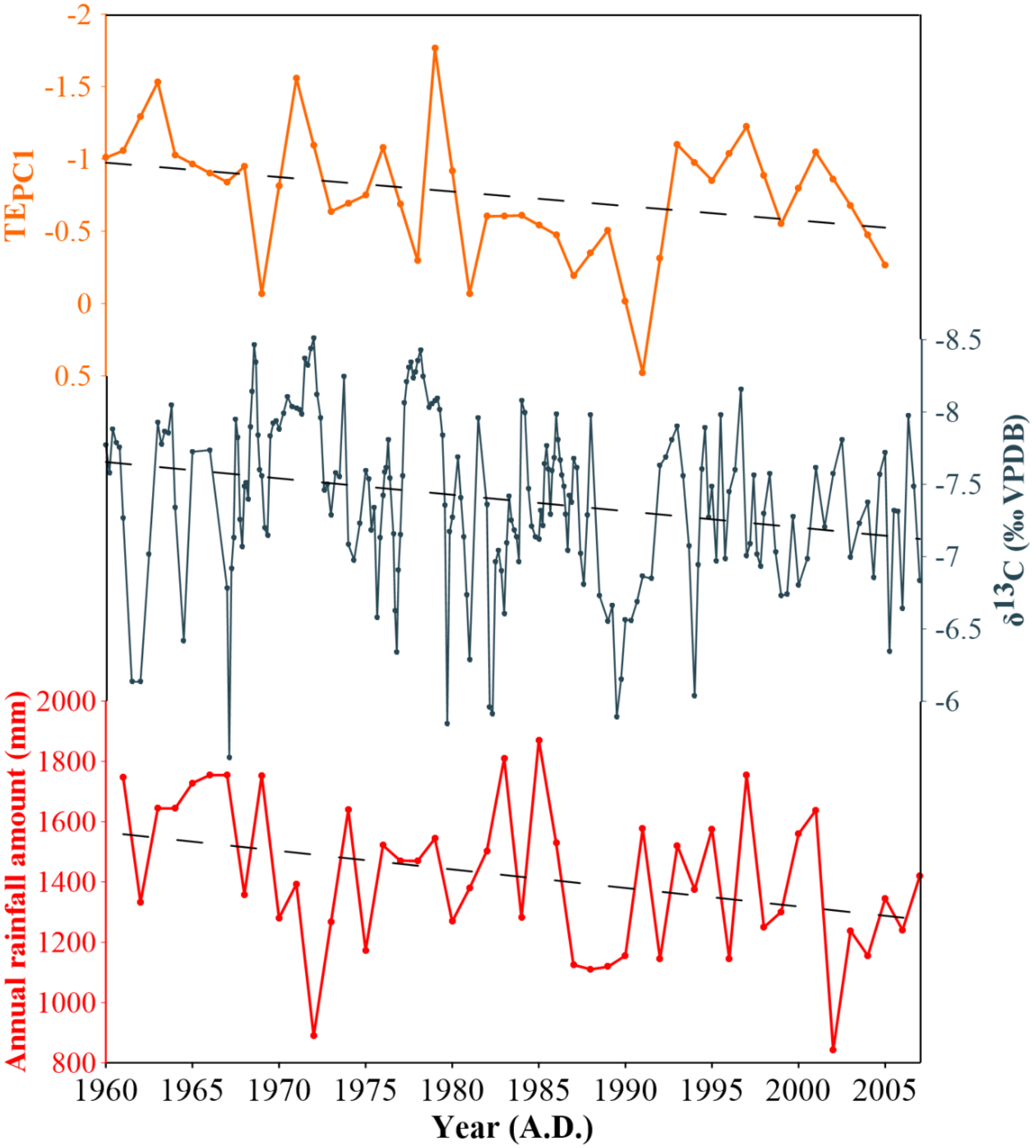

Fig. S17. Comparison between proxy reconstructions and instrumental data. Comparison between DG7 TE_{PC1} (orange), $\delta^{13}C$ (dark green), and instrumental annual rainfall amount data (red) from the Puan meteorological observatory, ~20 km to the northwest of Daoguan Cave. All data are linearly interpolated to produce a uniform 1-year temporal resolution, and then the calculated correlation coefficient (*97*). The correlation coefficient is -0.42 (5-year smoothed, $n = 43$, $p < 0.01$) between the $\delta^{13}C$ and annual rainfall amount and -0.43 (5-year smoothed, $n = 41$, $p < 0.01$) between the TE_{PC1} and annual rainfall amount. The dashed lines indicate the linear trend of the three records.

Table S1.

Table S1. ^{230}Th dating results for stalagmite DG7.

Sample Number	^{238}U (ppb)	^{232}Th (ppt)	^{230}Th/^{232}Th (atomic×10^{-6})	δ^{234}U* (measured)	^{230}Th / ^{238}U (activity)	^{230}Th Age (yr B.P.) (uncorrected)	^{230}Th Age (yr B.P.) (corrected)	$\delta^{234}U_{Initial}$** (corrected)
DG7-3	44.8± 0.1	3756 ± 11	8 ± 1	582.1 ± 4.5	0.0404 ± 0.0027	2811 ± 189	1262 ± 803	584.2 ± 4.7
DG7-135	35.7 ±0.1	1990 ± 5	6 ± 1	609.5 ± 3.9	0.0200 ± 0.0022	1363 ± 150	353 ± 529	610.1 ± 4.1

Errors are 2σ analytical errors.
U decay constants: $\lambda_{238}=1.55125\times10^{-10}$ (*103*) and $\lambda_{234}=2.82206\times10^{-6}y^{-1}$ (*75*). Th decay constant: $\lambda_{230}=9.1705\times10^{-6}$ ref. (*75*).
*$\delta^{234}U = ([^{234}U/^{238}U]_{activity} - 1) \times 1000$. **$\delta^{234}U_{initial}$ was calculated based on ^{230}Th age (T), i.e., $\delta^{234}U_{initial} = \delta^{234}U_{measured}\times e^{\lambda234\times T}$.
Corrected ^{230}Th ages assume the initial $^{230}Th/^{232}Th$ atomic ratio of $4.4\pm2.2\times10^{-6}$. Those are the values for a material at secular equilibrium, with the bulk earth $^{232}Th/^{238}U$ value of 3.8. The errors are arbitrarily assumed to be 50%.
B.P. stands for “Before Present” where the “Present” is defined as the year 1950 A.D.

Table S2.

Table S2. ^{210}Pb dating results for stalagmite DG7.

Sample Number	Weight (g)	Distance from top (mm)	Range (mm)	^{210}Pb (dpm/g)	Error (dpm/g)
DG7-1	0.1217	2.5	0-5	2.10	0.15
DG7-2	0.1327	12.5	10-15	0.92	0.08
DG7-3	0.1245	17.5	15-20	0.59	0.07
DG7-4	0.1755	27.5	25-30	0.49	0.05
DG7-5	0.1696	32.5	30-35	0.45	0.05
DG7-6	0.2229	37.5	35-40	0.33	0.04
DG7-7	0.2543	42.5	40-45	0.31	0.03
DG7-8	0.2014	47.5	45-50	0.27	0.03
DG7-9	0.2995	52.5	50-55	0.18	0.02
DG7-10	0.2907	57.5	55-60	0.16	0.02
DG7-11	0.3021	62.5	60-65	0.16	0.02

Table S3.

Table S3. Correlation between the cave $\delta^{18}O$ records in China

	Dongge Cave	Heshang Cave	Qujia Cave	Shihua Cave
Daoguan Cave (DG7)	0.37*	0.19*	0.31*	0.39*
Dongge Cave		0.35*	0.24*	0.51*
Heshang Cave			0.28*	0.59*
Qujia Cave				0.72*

*The correlation coefficient (97) is calculated with a 1-year equal interval interpolation of these records. Significant at the $p < 0.01$ level.

Table S4.

Table S4. Results of empirical mode decomposition (*104*) of the DG7 record.
Table S4a. Contribution rate of the IMF components over the past two centuries.

$\delta^{18}O$	IMF1	IMF2	IMF3	IMF4	**IMF5**	IMF6	IMF7	IMF8
Period (yr)	0.6	1.2	2.5	4.8	**10.3**	19.2	48.6	78.3
Contribution (%)	5.9%	9.6%	13.9%	19.9%	**29.5%**	12.8%	8.1%	0.4%
Ranking	7	5	3	2	**1**	4	6	8

Table S4b. Contribution rate of the IMF components between 1870 and 1950 A.D.

$\delta^{18}O$	IMF1	IMF2	IMF3	IMF4	IMF5	IMF6
Period (yr)	0.5	1.2	**2.6**	**4.8**	8.5	16.8
Contribution (%)	12.4%	26.0%	**25.2%**	**14.7%**	12.8%	8.8%
Ranking	5	1	**2**	**3**	4	6

Table S5.

Table S5. Correlation (*97*) between the stable isotope and trace element data from 1787 – 2005 (n=129, 3-point running mean). The principal component analysis (PCA) was performed on Mg/Ca, Sr/Ca, and Ba/Ca records. The result shows that the TE_{PC1}, accounting for 68.3 % of the total variance, can represent a leading mode of these trace element records. The PCA method can be referred to: http://www.geog.leeds.ac.uk/courses/other/statistics/spss/principalcomp/.

	$\delta^{13}C$	Mg/Ca	Sr/Ca	Ba/Ca	TE_{PC1}	$\delta^{18}O$
$\delta^{13}C$		0.47*	0.45*	0.27*	0.48*	-0.31*
Mg/Ca			0.66*	0.60*	0.82*	-0.35*
Sr/Ca				0.70*	0.88*	-0.38*
Ba/Ca					0.85*	-0.32*
TE_{PC1}						-0.42*
**$\delta^{13}C^a$**		**0.67***	**0.68***	**0.51***	**0.74***	**-0.33***

* Significant at the $p < 0.01$ level

$\delta^{13}C^a$ Removing the interval of $\delta^{13}C$ anomalies from AD 1941–1959.

REFERENCES

1. Y. Ding, Z. Wang, Y. Sun, Inter-decadal variation of the summer precipitation in East China and its association with decreasing Asian summer monsoon. Part I: Observed evidences. *Int. J. Climatol.* **28**, 1139–1161 (2008).

2. Y. Ding, Y. Sun, Y. Liu, D. Si, Z. Wang, Y. Zhu, Y. Liu, Y. Song, J. Zhang, Interdecadal and interannual variabilities of the Asian summer monsoon and its projection of future change. *Chin. J. Atmos. Sci.* **37**, 253–280 (2013).

3. Y. Ding, D. Si, Y. Liu, Z. Wang, Y. Li, L. Zhao, Y. Song, On the characteristics, driving forces and inter-decadal variability of the East Asian summer monsoon. *Chin. J. Atmos. Sci.* **42**, 533–558 (2018).

4. H. Douville, K. Raghavan, J. Renwick, R. P. Allan, P. A. Arias, M. Barlow, R. Cerezo-Mota, A. Cherchi, T. Y. Gan, J. Gergis, D. Jiang, A. Khan, W. Pokam Mba, D. Rosenfeld, J. Tierney, O. Zolina, *Climate Change 2021: The Physical Science Basis. Contribution of Working Group I to the Sixth Assessment Report of the Intergovernmental Panel on Climate Change* (Cambridge Univ. Press, 2021).

5. P. Zhang, H. Cheng, R. L. Edwards, F. Chen, Y. Wang, X. Yang, J. Liu, M. Tan, X. Wang, J. Liu, C. An, Z. Dai, J. Zhou, D. Zhang, J. Jia, L. Jin, K. R. Johnson, A test of climate, sun, and culture relationships from an 1810-year Chinese cave record. *Science* **322**, 940–942 (2008).

6. X. Li, H. Cheng, L. Tan, F. Ban, A. Sinha, W. Duan, H. Li, H. Zhang, Y. Ning, G. Kathayat, R. L. Edwards, The East Asian summer monsoon variability over the last 145 years inferred from the Shihua Cave record, North China. *Sci. Rep.* **7**, 7078 (2017).

7. J. Zhao, H. Cheng, J. Cao, A. Sinha, X. Dong, L. Pan, C. Pérez-Mejías, H. Zhang, H. Li, J. Wang, K. Wang, J. Cui, Y. Yang, Orchestrated decline of Asian summer monsoon and Atlantic meridional overturning circulation in global warming period. *Innov. Geosci.* **1**, 100011 (2023).

8. C. Jin, J. Liu, B. Wang, M. Yan, L. Ning, Decadal variations of the East Asian summer monsoon forced by the 11-year insolation cycle. *J. Climate* **32**, 2735–2745 (2019).

9. L. Zhao, J. Wang, Z. Xiao, Y. Ding, W. Huo, J. Liu, Solar 11-year cycle-modulated north-south contrasting patterns of summer precipitation in China. *J. Climate* **38**, 3277–3294 (2025).

10. Q. Yang, Z. Ma, X. Fan, Z. Yang, Z. Xu, P. Wu, Decadal modulation of precipitation patterns over eastern China by sea surface temperature anomalies. *J. Climate* **30**, 7017–7033 (2017).

11. J. Zhu, K. Zhao, Y. Wang, Y. Cui, Y. Liang, H. Cheng, R. L. Edwards, X. Kong, X. Shao, S. Chen, L. Pang, Decadal modulation of East Asian summer monsoon variations by external forcing and internal variability. *Quat. Sci. Rev.* **293**,107720 (2022).

12. W. Sun, D. Chen, G. Lü, L. Ning, C. Gao, R. Zhang, B. Wang, J. Liu, Impacts of major volcanic eruptions over the past two millennia on both global and Chinese climates: A review. *Sci. China Earth Sci.* **67**, 61–78 (2024).

13. Y. Wang, H. Cheng, R. L. Edwards, Y. He, X. Kong, Z. An, J. Wu, M. J. Kelly, C. A. Dykoski, X. Li, The Holocene Asian monsoon: Links to solar changes and North Atlantic climate. *Science* **308**, 854–857 (2005).

14. Q. Ge, *Climate Changes in Dynastic China* (Science Press, 2010).

15. W. Sun, B. Wang, J. Liu, D. A. Bello, U. Büntgen, E. Xoplaki, D. Chen, H. Shi, N. Di Cosmo, Impact of the centennial changes in ENSO on the rise of the Chinese Qing empire. *NPJ Clim. Atmos. Sci.* **8**, 61 (2025).

16. Q. Ge, W. Wang, Population pressure, climate change and Taiping Rebellion. *Geogr. Res.* **14**, 32–41 (1995).

17. C. Jiang, W. Qiang, H. F. Lee, Exploring the spatial interplay between hydro-climatic extremes and armed conflicts in history. *NPJ Herit. Sci.* **13**, 209 (2025).

18. W. Zhang, K. Furtado, P. Wu, T. Zhou, R. Chadwick, C. Marzin, J. Rostron, D. Sexton, Increasing precipitation variability on daily-to-multiyear time scales in a warmer world. *Sci. Adv.* **7**, eabf8021 (2021).

19. W. Zhang, T. Zhou, P. Wu, Anthropogenic amplification of precipitation variability over the past century. *Science* **385**, 427–432 (2024).

20. N. He, J. Yin, P. Liu, X. Fu, Q. Zhang, L. Cheng, S. Moulds, A. Volchak, Global increases in dry-wet abrupt alternation events under climate change. *Geophys. Res. Lett.* **52**, e2025GL117322 (2025).

21. K. R. Johnson, C. Hu, N. S. Belshaw, G. M. Henderson, Seasonal trace-element and stable-isotope variations in a Chinese speleothem: The potential for high-resolution paleomonsoon reconstruction. *Earth Planet. Sci. Lett.* **244**, 394–407 (2006).

22. M. Ji, J. Shen, E. Zhang, Y. Li, R. Chen, Biomass burning patterns around Lake Xingyun during the last 137 years based on a high-resolution analysis of macroscopic char coal of lake sediment. *Quat. Sci.* **38**, 963–970 (2018).

23. C. Huang, L. Lu, Y. Li, Y. He, N. Shang, Y. Bai, H. Yu, T. Huang, A. Zhu, H. Yang, K. Zhao, Y. Yu, Anthropogenic-driven alterations in black carbon sequestration and the structure in a deep plateau lake. *Environ. Sci. Technol.* **55**, 6467–6475 (2021).

24. C. H. Hendy, The isotopic geochemistry of speleothems-I. The calculation of the effects of different modes of formation on the isotopic composition of speleothems and their applicability as palaeoclimatic indicators. *Geochim. Cosmochim. Acta* **35**, 801–824 (1971).

25. J. A. Dorale, Z. Liu, Limitations of Hendy test criteria in judging the paleoclimatic suitability of speleothems and the need for replication. *J. Cave Karst Stud.* **71**, 73–80 (2009).

26. B. L. Otto-Bliesner, E. C. Brady, J. Fasullo, A. Jahn, L. Landrum, S. Stevenson, N. Rosenbloom, A. Mai, G. Strand, Climate variability and change since 850 CE: An ensemble approach with the community Earth system model. *Bull. Am. Meteorol. Soc.* **97**, 735–754 (2016).

27. S. Stevenson, B. L. Otto-Bliesner, E. C. Brady, J. Nusbaumer, C. Tabor, R. Tomas, D. C. Noone, Z. Liu, Volcanic eruption signatures in the isotope-enabled Last Millennium Ensemble. *Paleoceanogr. Paleoclimatol.* **34**, 1534–1552 (2019).

28. H. Cheng, R. L. Edwards, A. Sinha, C. Spötl, L. Yi, S. Chen, M. Kelly, G. Kathayat, X. Wang, X. Li, X. Kong, Y. Wang, Y. Ning, H. Zhang, The Asian monsoon over the past 640,000 years and ice age terminations. *Nature* **534**, 640–646 (2016).

29. Y. Liang, K. Zhao, R. L. Edwards, Y. Wang, Q. Shao, Z. Zhang, B. Zhao, Q. Wang, H. Cheng, X. Kong, East Asian monsoon changes early in the last deglaciation and insights into the interpretation of oxygen isotope changes in the Chinese stalagmite record. *Quat. Sci. Rev.* **250**, 106699 (2020).

30. Y. Wang, H. Cheng, R. L. Edwards, Z. An, J. Wu, C. Shen, J. A. Dorale, A high-resolution absolute-dated late Pleistocene monsoon record from Hulu Cave, China. *Science* **294**, 2345–2348 (2001).

31. D. Yuan, H. Cheng, R. L. Edwards, C. A. Dykoski, M. J. Kelly, M. Zhang, J. Qing, Y. Lin, Y. Wang, J. Wu, J. A. Dorale, Z. An, Y. Cai, Timing, duration, and transitions of the last interglacial asian monsoon. *Science* **304**, 575–578 (2004).

32. K. Zhao, Y. Wang, R. L. Edwards, H. Cheng, D. Liu, X. Kong, A high-resolved record of the Asian Summer Monsoon from Dongge Cave, China for the past 1200 years. *Quat. Sci. Rev.* **122**, 250–257 (2015).

33. M. Wang, C. Hu, Y. Liu, L. Li, S. Xie, K. R. Johnson, Precipitation in eastern China over the past millennium varied with large-scale climate patterns. *Commun. Earth Environ.* **3**, 321 (2022).

34. F. S. R. Pausata, D. S. Battisti, K. H. Nisancioglu, C. M. Bitz, Chinese stalagmite $\delta^{18}O$ controlled by changes in the Indian monsoon during a simulated Heinrich event. *Nat. Geosci.* **4**, 474–480 (2011)

35. G. Liu, X. Li, H. W. Chiang, H. Cheng, S. Yuan, S. Chawchai, S. He, Y. Lu, L. T. Aung, P. M. Maung, W. N. Tun, K. M. Oo, X. Wang, On the glacial-interglacial variability of the Asian monsoon in speleothem $\delta^{18}O$ records. *Sci. Adv.* **6**, eaay8189 (2020).

36. Z. Liu, X. Wen, E. C. Brady, B. L. Otto-Bliesner, G. Yu, H. Lu, H. Cheng, Y. Wang, W. Zheng, Y. Ding, R. L. Edwards, J. Cheng, W. Liu, H. Yang, Chinese cave records and the East Asia Summer Monsoon. *Quat. Sci. Rev.* **83**, 115–128 (2014).

37. C. He, Z. Liu, B. L. Otto-Bliesner, E. C. Brady, C. Zhu, R. Tomas, P. U. Clark, J. Zhu, A. Jahn, S. Gu, J. Zhang, J. Nusbaumer, D. Noone, H. Cheng, Y. Wang, M. Yan, Y. Bao, Hydroclimate footprint of pan-Asian monsoon water isotope during the last deglaciation. *Sci. Adv.* **7**, eabe2611 (2021).

38. Y. Liu, Y. Ding, Analysis and numerical simulation of the teleconnection between Indian summer monsoon and precipitation in North China. *Acta. Meteor. Sin.* **66**, 789–799 (2008).

39. C. Qin, B. Yang, A. Bräuning, F. Charpentier Ljungqvist, T. J. Osborn, V. Shishov, M. He, S. Kang, L. Schneider, J. Esper, U. Büntgen, J. Grießinger, D. Huang, P. Zhang, S. Talento, E. Xoplaki, J. Luterbacher, N. C. Stenseth, Persistent humid climate favored the Qin and Western Han Dynasties in China around 2,200 y ago. *Proc. Natl. Acad. Sci. U.S.A.* **122**, e2415294121 (2025).

40. M. K. Roxy, K. Ritika, P. Terray, R. Murtugudde, K. Ashok, B. N. Goswami, Drying of Indian subcontinent by rapid Indian Ocean warming and a weakening land-sea thermal gradient. *Nat. Commun.* **6**, 7423 (2015).

41. T. Zhou, W. Zhang, L. Zhang, X. Zhang, Y. Qian, D. Peng, S. Ma, B. Dong, The dynamic and thermodynamic processes dominating the reduction of global land monsoon precipitation driven by anthropogenic aerosols emission. *Sci. China Earth Sci.* **63**, 919–933 (2020).

42. H. E. Ridley, Y. Asmerom, J. U. L. Baldini, S. F. M. Breitenbach, V. V. Aquino, K. M. Prufer, B. J. Culleton, V. Polyak, F. A. Lechleitner, D. J. Kennett, M. Zhang, N. Marwan, C. G. Macpherson, L. M. Baldini, T. Xiao, J. L. Peterkin, J. Awe, G. H. Haug, Aerosol forcing of the position of the intertropical convergence zone since AD 1550. *Nat. Geosci.* **8**, 195–200 (2015).

43. C. Da, X. Wang, W. Sun, J. Liu, L. Ning, G. Chen, Decadal variability in $\delta^{18}O$ over the East Asian monsoon region responding to solar activity over the last millennium. *Sci. China Earth Sci.* **68**, 2853–2866 (2025).

44. G. A. Meehl, J. M. Arblaster, K. Matthes, F. Sassi, H. van Loon, Amplifying the Pacific climate system response to a small 11-year solar cycle forcing. *Science* **325**, 1114–1118 (2009).

45. K. M. Cobb, C. D. Charles, H. Cheng, R. L. Edwards, El Niño/Southern Oscillation and tropical Pacific climate during the last millennium. *Nature* **424**, 271–276 (2003).

46. S. C. Sanchez, N. Westphal, G. H. Haug, H. Cheng, R. L. Edwards, T. Schneider, K. M. Cobb, C. D. Charles, A continuous record of central tropical pacific climate since the midnineteenth century reconstructed from fanning and palmyra island corals: A case study in coral data reanalysis. *Paleoceanogr. Paleoclimatol.* **35**, e2020PA003848 (2020).

47. J. Li, S. Xie, E. R. Cook, M. S. Morales, D. A. Christie, N. C. Johnson, F. Chen, R. D'Arrigo, A. M. Fowler, X. Gou, K. Fang, El Niño modulations over the past seven centuries. *Nat. Clim. Change* **3**, 822–826 (2013).

48. R. Muscheler, F. Joos, J. Beer, S. A. Müller, M. Vonmoos, I. Snowball, Solar activity during the last 1000 yr inferred from radionuclide records. *Quat. Sci. Rev.* **26**, 82–97 (2007).

49. E. R. Thomas, E. W. Wolff, R. Mulvaney, J. P. Steffensen, S. J. Johnsen, C. Arrowsmith, J. W. C. White, B. Vaughn, T. Popp, The 8.2 kyr event from Greenland ice cores. *Quat. Sci. Rev.* **26**, 70–81 (2007).

50. Z. Liu, W. Zhou, X. Wang, Extreme meteorological drought events over China (1951–2022): Migration pattern, diversity of temperature extremes, and decadal variations. *Adv. Atmos. Sci.* **41**, 2313–2336 (2024).

51. Chinese Academy of Meteorological Sciences, *Yearly Charts of Dryness/Wetness in China for the Last 500-Year Period* (Sinomaps Press, 1981).

52. Q. Cai, H. Zhang, Y. Liu, M. Xie, M. Ren, Q. Li, C. Sun, H. Song, R. Liu, K. Meng, D. Chen, Tree-ring $\delta^{18}O$, a window into the summer hydroclimatic variations in the Central Water Tower of China. *J. Hydrol.* **664**, 134466 (2026).

53. A. Sinha, G. Kathayat, H. Cheng, S. F. M. Breitenbach, M. Berkelhammer, M. Mudelsee, J. Biswas, R. L. Edwards, Trends and oscillations in the Indian summer monsoon rainfall over the last two millennia. *Nat. Commun.* **6**, 6309 (2015).

54. G. Kathayat, A. Sinha, S. F. M. Breitenbach, L. Tan, C. Spötl, H. Li, X. Dong, H. Zhang, Y. Ning, R. J. Allan, V. Damodaran, R. L. Edwards, H. Cheng, Protracted Indian monsoon droughts of the past millennium and their societal impacts. *Proc. Natl. Acad. Sci. U.S.A.* **119**, e2207487119 (2022).

55. Y. Asmerom, V. J. Polyak, J. B. T. Rasmussen, S. J. Burns, M. Lachniet, Multidecadal to multicentury scale collapses of Northern Hemisphere monsoons over the past millennium. *Proc. Natl. Acad. Sci. U.S.A.* **110**, 9651–9656 (2013).

56. T. M. Shanahan, J. T. Overpeck, K. J. Anchukaitis, J. W. Beck, J. E. Cole, D. L. Dettman, J. A. Peck, C. A. Scholz, J. W. King, Atlantic forcing of persistent drought in West Africa. *Science* **324**, 377–380 (2009).

57. G. Bond, B. Kromer, J. Beer, R. Muscheler, M. N. Evans, W. Showers, S. Hoffmann, R. Lotti-Bond, I. Hajdas, G. Bonani, Persistent solar influence on North Atlantic climate during the Holocene. *Science* **294**, 2130–2136 (2001).

58. L. Caesar, S. Rahmstorf, A. Robinson, G. Feulner, V. Saba, Observed fingerprint of a weakening Atlantic Ocean overturning circulation. *Nature* **556**, 191–196 (2018).

59. S. Rahmstorf, J. E. Box, G. Feulner, M. E. Mann, A. Robinson, S. Rutherford, E. J. Schaffernicht, Exceptional twentieth-century slowdown in Atlantic Ocean overturning circulation. *Nat. Clim. Change* **5**, 475–480 (2015).

60. D. J. R. Thornalley, D. W. Oppo, P. Ortega, J. I. Robson, C. M. Brierley, R. Davis, I. R. Hall, P. Moffa-Sanchez, N. L. Rose, P. T. Spooner, I. Yashayaev, L. D. Keigwin, Anomalously weak

Labrador Sea convection and Atlantic overturning during the past 150 years. *Nature* **556**, 227–230 (2018).

61. D. Si, Y. Ding, Oceanic forcings of the interdecadal variability in East Asian summer rainfall. *J. Climate* **29**, 7633–7649 (2016).

62. Y. Zhang, J. M. Wallace, D. S. Battisti, ENSO-like interdecadal variability: 1900–93. *J. Climate* **10**, 1004–1020 (1997).

63. M. E. Mann, Z. Zhang, S. Rutherford, R. S. Bradley, M. K. Hughes, D. Shindell, C. Ammann, G. Faluvegi, F. Ni, Global signatures and dynamical origins of the Little Ice Age and medieval climate anomaly. *Science* **326**, 1256–1260 (2009).

64. D. B. Enfield, A. M. Mestas-Nunez, P. J. Trimble, The Atlantic Multidecadal Oscillation and its relationship to rainfall and river flows in the continental U.S. *Geophys. Res. Lett.* **28**, 2077–2080 (2001).

65. F. Lapointe, R. S. Bradley, P. Francus, N. L. Balascio, M. B. Abbott, J. S. Stoner, G. St-Onge, A. De Coninck, T. Labarre, Annually resolved Atlantic sea surface temperature variability over the past 2,900 y. *Proc. Natl. Acad. Sci. U.S.A.* **117**, 27171–27178 (2020).

66. H. F. Lee, D. D. Zhang, A tale of two population crises in recent Chinese history. *Clim. Change* **116**, 285–308 (2013).

67. H. F. Lee, Climate-induced agricultural shrinkage and overpopulation in late imperial China. *Climate Res.* **59**, 229–242 (2014).

68. W. Gao, *The History of Natural Disasters in China* (Seismological Press, 1997).

69. W. Li, *China's Modern Agricultural History Materials (First Series)* (SDX Joint Publishing Company, 1957).

70. Q. Xie, W. Hu, *Guang Xi Tong Zhi (Guangxi Province Gazetteer)* (Guangxi People's Publishing House, 1988).

71. D. Li, C. Shi, *Veritable Records of the Qing Dynasty in Daoguang Period* (Zhonghua Book Company, 1985).

72. Y. Guo, *Historical Charts of the Taiping Rebellion* (Sinomaps Press, 1988).

73. A. Feuerwerker, S. Chiang, The nien rebellion. *Harv. J. Asiat. Stud.* **19**, 165–167 (1956).

74. Z. Wei, X. Fang, Y. Su, Climate change, fiscal balance and dynastical cycles in China over the past 2000 years. *Quat. Sci.* **40**, 1180–1192 (2020).

75. H. Cheng, R. L. Edwards, C. Shen, V. J. Polyak, Y. Asmerom, J. Woodhead, J. Hellstrom, Y. Wang, X. Kong, C. Spötl, X. Wang, E. C. Alexander Jr., Improvements in ^{230}Th dating, ^{230}Th and ^{234}U half-life values, and U–Th isotopic measurements by multi-collector inductively coupled plasma mass spectrometry. *Earth Planet. Sci. Lett.* **371**, 82–91 (2013).

76. D. A. Richards, J. A. Dorale, Uranium-series chronology and environmental applications of speleothems. *Rev. Mineral. Geochem.* **52**, 407–460 (2003).

77. J. Hellstrom, U–Th dating of speleothems with high initial ^{230}Th using stratigraphical constraint. *Quat. Geochronol.* **1**, 289–295 (2006).

78. M. Baskaran, T. M. Iliffe, Age determination of recent cave deposits using excess ^{210}Pb–A new technique. *Geophys. Res. Lett.* **20**, 603–606 (1993).

79. J. A. Dorale, R. L. Edwards, E. Ito, L. A. González, Climate and vegetation history of the midcontinent from 75 to 25 ka: A speleothem record from Crevice Cave, Missouri, USA. *Science* **282**, 1871–1874 (1998).

80. F. Mcdermott, Palaeo-climate reconstruction from stable isotope variations in speleothems: A review. *Quat. Sci. Rev.* **23**, 901–918 (2004).

81. L. Rong, S. Wang, X. Du, Responses of δ^{13}C values of plant leaves to environmental gradients along environmental gradient factors in rocky desertified area of a typical karst Gorge. *Environ. Sci.* **29**, 2885–2893 (2008).

82. X. Du, S. Wang, Seasonal variations and responses to different rocky desertification degrees of foliar $\delta^{13}C$ values of 5 local plant species in karst areas. *Earth Environ.* **38**, 129–137 (2010).

83. W. Luo, S. Wang, X. Xie, Y. Zhou, T. Li, Stable carbon isotope variations in cave percolation waters and their implications in four caves of Guizhou, China. *Acta Geol. Sin-Engl.* **87**, 1396–1411 (2013).

84. T. Li, C. Huang, L. Tian, M. Suarez, Y. Gao, Variation of $\delta^{13}C$ in plant-soil-cave systems in karst regions with different degrees of rocky desertification in southwest China and implications for paleoenvironment reconstruction. *J. Cave Karst Stud.* **80**, 212–228 (2018).

85. F. He, Q. Ge, J. Dai, S. Lin, Quantitative analysis on forest dynamics of China in recent 300 years. *Acta Geogr. Sin.* **62**, 30–40 (2007).

86. Editorial Committee of Local Annals in Guizhou Province, *Annals of Guizhou province • Annals of Industry and Economy.* (Guizhou People's Publishing House, 2003).

87. L. Meng, "Black carbon burial in Chinese lakes: Characteristics, drivers, and carbon sink significance," thesis, Nanjing Normal University, Nanjing (2025).

88. I. J. Fairchild, C. L. Smith, A. Baker, L. Fuller, C. Spötl, D. Mattey, F. McDermott, Modification and preservation of environmental signals in speleothems. *Earth Sci. Rev.* **75**, 105–153 (2006).

89. I. J. Fairchild, P. C. Treble, Trace elements in speleothems as recorders of environmental change. *Quat. Sci. Rev.* **28**, 449–468 (2009).

90. D. J. Sinclair, J. L. Banner, F. W. Taylor, J. Partin, J. Jenson, J. Mylroie, E. Goddard, T. Quinn, J. Jocson, B. Miklavič, Magnesium and strontium systematics in tropical speleothems from the Western Pacific. *Chem. Geol.* **294-295**, 1–17 (2012).

91. M. L. Griffiths, A. K. Kimbrough, M. K. Gagan, R. N. Drysdale, J. E. Cole, K. R. Johnson, J. Zhao, B. I. Cook, J. C. Hellstrom, W. S. Hantoro, Western Pacific hydroclimate linked to global climate variability over the past two millennia. *Nat. Commun.* **7**, 11719 (2016).

92. H. Zhang, M. L. Griffiths, J. C. H. Chiang, W. W. Kong, S. T. Wu, A. Atwood, J. Huang, H. Cheng, Y. Ning, S. Xie, East Asian hydroclimate modulated by the position of the westerlies during Termination I. *Science* **362**, 580–583 (2018).

93. E. Kalnay, M. Kanamitsu, R. Kistler, W. Collins, D. Deaven, L. Gandin, M. Iredell, S. Saha, G. White, J. Woollen, Y. Zhu, A. Leetmaa, R. Reynolds, The NCEP/NCAR 40-year reanalysis project. *Bull. Am. Meteorol. Soc.* **77**, 437–471 (1996).

94. J. B. Liu, J. H. Chen, X. J. Zhang, Y. Li, Z. G. Rao, F. H. Chen, Holocene East Asian summer monsoon records in northern China and their inconsistency with Chinese stalagmite $\delta^{18}O$ records. *Earth Sci. Rev.* **148**, 194–208 (2015).

95. R. R. Draxler, G. D. Hess, An overview of the HYSPLIT_4 modelling system for trajectories. *Aust. Met. Mag.* **47**, 295–308 (1998).

96. A. Baker, C. L. Smith, C. Jex, I. J. Fairchild, D. Genty, L. Fuller, Annually laminated speleothems: A review. *Int. J. Speleol.* **37**, 193–206 (2008).

97. M. Mudelsee, Estimating Pearson's correlation coefficient with bootstrap confidence interval from serially dependent time series. *Math. Geol.* **35**, 651–665 (2003).

98. R. Wilson, K. Anchukaitis, K. R. Briffa, U. Büntgen, E. Cook, R. D'Arrigo, N. Davi, J. Esper, D. Frank, B. Gunnarson, G. Hegerl, S. Helama, S. Klesse, P. J. Krusic, H. W. Linderholm, V. Myglan, T. J. Osborn, M. Rydval, L. Schneider, A. Schurer, G. Wiles, P. Zhang, E. Zorita, Last millennium northern hemisphere summer temperatures from tree rings: Part I: The long term context. *Quat. Sci. Rev.* **134**, 1–18 (2016).

99. Q. Ge, Z. Hao, J. Zheng, X. Shao, Temperature changes over the past 2000 yr in China and comparison with the Northern Hemisphere. *Clim. Past* **9**, 1153–1160 (2013).

100. M. E. Mann, M. A. Cane, S. E. Zebiak, A. Clement, Volcanic and solar forcing of the tropical pacific over the past 1000 years. *J. Climate* **18**, 417–456 (2005).

101. I. J. Fairchild, A. Baker, *Speleothem Science: From Process to Past Environments* (John Wiley & Sons, 2012).

102. Y. Huang, I. J. Fairchild, Partitioning of Sr^{2+} and Mg^{2+} into calcite under karst-analogue experimental conditions. *Geochim. Cosmochim. Acta* **65**, 47–62 (2001).

103. A. H. Jaffey, K. F. Flynn, L. E. Glendenin, W. C. Bentley, A. M. Essling, Precision measurement of half-lives and specific activities of ^{235}U and ^{238}U. *Phys. Rev. C* **4**, 1889–1906 (1971).

104. Z. Wu, N. Huang, S. Long, C. Peng, On the trend, detrending, and variability of nonlinear and nonstationary time series. *Proc. Natl. Acad. Sci. U.S.A.* **104**, 14889–14894 (2007).
